# Sensitivity of machine learning regression models to data structure and quality in crop yield prediction

**DOI:** 10.1371/journal.pone.0353938

**Published:** 2026-09-01

**Authors:** Mahoukpégo Luc Zinzinhédo, Mèvognon Firmin Mitchozounnou, Kolawolé Valère Salako, Romain Glèlè Kakaï

**Affiliations:** Laboratoire de Biomathématiques et d’Estimations Forestières, Faculté des Sciences Agronomiques, Université d’Abomey-Calavi, Abomey-Calavi, Atlantique, Bénin; World Health Organization, Regional Office for South-East Asia, INDIA

## Abstract

Accurate crop yield prediction is essential for agricultural planning, yet machine learning (ML) models remain highly sensitive to the quality and structure of input data. This study uses simulated datasets to systematically investigate how data structure (sample size and number of predictors), data imperfections (missing values and multicollinearity), and pre-processing methods (imputation techniques and principal component analysis) influence ML regression performance. The performance of ML-based models (RF, SVM, MLR, XGBoost, LightGBM, NNet, and kNN) on the pre-processed data was evaluated. In total, each algorithm was tested on 1,728 datasets and pre-processing scenarios, yielding 12,096 model-scenario evaluations across the seven ML algorithms. Results show that missing data decreases performance (*R*^2^ drops by up to 20.63%; MAE increases by 12.67%), while multicollinearity may inflate *R*^2^ values despite poorer MAE performance. Larger sample sizes consistently improve prediction accuracy (*R*^2^ = +18.87%; MAE = −8.16%), whereas more predictors generally reduce it (*R*^2^ = −8.47%; MAE = +44.74%). Regression-based imputation improved both *R*^2^ and MAE the most, while RF demonstrated greater robustness across varying conditions. These simulation findings were further validated on five real-world crop datasets (Maize, Yam, Cassava, Sorghum, and Peanuts), confirming that despite RF remains the safest and most robust default, no single pre-processing strategy is universally optimal and that the selection of imputation method and dimensionality reduction must be strictly contingent upon the dataset’s specific missingness rate and correlation structure. Overall, this study highlights the complex interplay between data characteristics and pre-processing, urging the development of clearer guidelines for applying ML to agricultural datasets.

## Introduction

In statistical modeling, real-world datasets often present a variety of imperfections (such as multicollinearity, limited sample sizes, missing values, outliers, or duplicate records) that significantly challenge robust analysis [1,2]. With such a dataset, statistical analysis is not straightforward, and the results are likely to be biased [3]. These imperfections are pervasive across disciplines, including medicine [4–6], epidemiology [7,8], agronomy [9–11], psychology [12], geography [13], astronomy [14], water science [15], etc. Performing a statistical analysis with an imperfect dataset, without any pre-processing, satisfies the *garbage in, garbage out* (GIGO) of George Fuechsel [16,17]. Therefore, the management of imperfections in datasets deserves particular attention.

Pre-processing refers to a set of techniques aimed at improving data quality before analysis. This includes imputation of missing values, normalization, and feature extraction or reduction. Yet, the choice of any of these techniques is still subjective. For example, in crop yield prediction (CYP), while some studies lack data to achieve better yield prediction [18,19], some eliminate data points with missing values and outliers [20,21] and others impute them with median [22], Expectation–Maximization technique [23], moving average method also know as arithmetic average or mean method [24], regression [25], etc. Other imputation methods include linear interpolation [26], average imputation by nearby districts, k-nearest neighbor [27], random forest-based method [28], multiple imputation by chained equations [29], etc. To improve the accuracy in statistical analysis, several studies evaluate and propose methods for data pre-processing. For instance, to deal with uncertain, imprecise, or missing values, several approaches have been proposed, among which the fuzzy K-nearest neighbor classifier developed by [30] and a nonparametric iterative imputation algorithm [31]. [25] found that among classical statistical imputation methods like Arithmetic Average Replacement, Median Imputation, Linear Interpolation, and Average Imputation by Nearby Districts, the Arithmetic Average Replacement method provides better results, while among Machine learning-based imputations methods like: k-nearest neighbor, Random Forest-based method, linear regression, and the Multiple Imputation by Chained Equations, the Random Forest-based method is better.

Similarly, data normalization is a common pre-processing technique. While the true scale is used in some studies [32,33], other studies rescale predictors using several techniques such as min-max [34,35] and z-score [36,37]. Moreover, when extracting information from a dataset, principal components are also employed [38,39] for instance, to address multicollinearity [40].

Pre-processing decisions have been shown to profoundly affect Machine Learning (ML) performance. For example, [41] investigated how feature selection, feature extraction, and their interaction affect ML performance in predicting crop yield. Findings show that models perform better when both feature selection and feature extraction are applied, instead of only one of them. Similarly, [15] examined the impact of the variational mode decomposition, wavelet packet decomposition, complete ensemble empirical mode decomposition with adaptive noise, extreme-point symmetric mode decomposition, and singular spectrum analysis on the performance of the gated recurrent unit network. They found that the variational mode decomposition and the wavelet packet decomposition perform better than the others, indicating that the models perform differently in different conditions. The number of predictor variables is also critical for the performance of the ML. Some models are based on a large number of features: more than 100 (e.g., 160 – [42], 165 – [43]), while others are based on fewer than 20 features (3 – [44], 18 – [32]). This diversity in the choice of methods can substantially affect the results.

The same challenge is real in health science. For instance, when predicting diabetes disease cases: classical baselines were outperformed in handling imperfect data [45,46]. Indeed, in diabetes prediction, missing values, limited labeled data, high-dimensional feature spaces, and outliers have been identified as primary obstacles to reliable predictive performance, challenges that are structurally analogous to those encountered in agricultural yield prediction. Unlike clinical settings where data collection protocols are relatively standardised, however, field trial data are further characterised by spatial heterogeneity, seasonal variability, and low observation-to-predictor ratios, making the consequences of suboptimal pre-processing choices considerably more severe and less predictable. Despite this growing body of work, the field lacks a systematic understanding of how data structure (e.g., sample size, number of predictors), imperfections (e.g., missing data, multicollinearity), and pre-processing methods interact to influence ML model performance. Few studies have simultaneously considered the combined effect of these factors, making it unclear which ML algorithms are most robust under different conditions.

Data pre-processing techniques were demonstrated to significantly improve ML-based models, and some may decrease the models’ performance [47], meaning that an inappropriate choice of the pre-processing technique may lead to an inaccurate prediction. Similarly, several studies like [48] show the impact of number of variables, and propose data dimension reduction methods, including the principal component analysis. This simulation-based study addresses that gap. We assess the individual and joint effects of data structure, imperfections, and pre-processing on the predictive performance of seven ML models: random forest (RF), support vector machine (SVM), multiple linear regression (MLR), Extreme Gradient Boosting (XGBoost), Light Gradient Boosting Machine (LGBM), Neural Networks (NNet), and k-nearest neighbors (kNN). Using synthetic data based on real-world patterns, we simulate various scenarios involving different levels of missingness, multicollinearity, predictor counts, and sample sizes. We then apply a range of imputation techniques and principal component analysis (PCA) for pre-processing and evaluate the models through repeated random subsampling validation. This approach allows us to (i) Compare the robustness and performance of the selected ML algorithms; (ii) Quantify the sensitivity of model performance to variations in data structure and quality; (iii) Identify key interactions between pre-processing techniques and data characteristics. The results of this study will inform best practices in the pre-processing of imperfect datasets for ML-based prediction, especially in the context of crop yield modeling.

## Methods

**Fig 1:**
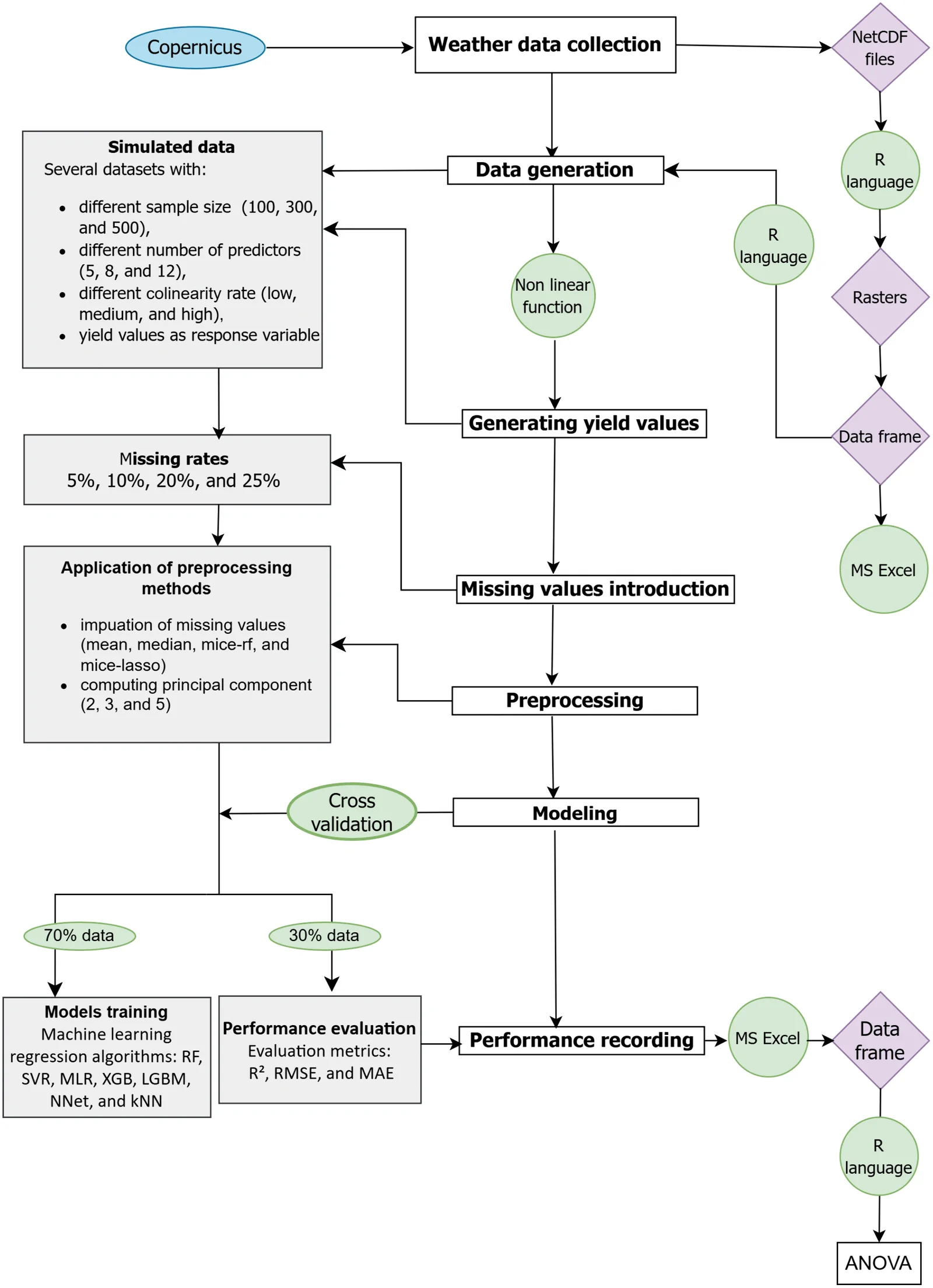
Simulation and evaluation workflow of ML performance under data imperfections.

The overall approach used is depicted in Fig 1. The simulation process was based on real data downloaded online (from Copernicus Climate Change Service). Missing values were introduced into the simulated data, and pre-processing methods were applied. After the pre-processing, a repeated random subsampling validation approach was used to train and test seven ML techniques, namely random forest (RF), support vector machine (SVM), multilinear regression (MLR), Extreme Gradient Boosting (XGBoost), Light Gradient Boosting Machine (LGBM), Neural Networks (NNet), and k-nearest neighbor (kNN).

## Simulation process

### Original data acquisition and pre-processing

The original data used were related to weather and were downloaded from the Copernicus Climate Change Service database (https://climate.copernicus.eu/) as NetCDF files. These files gather the bioclimatic variables (S1 Table), water vapor pressure, and wind speed, covering the period from 1995 to 2020 worldwide. They were imported into the R programming language for pre-processing. We transformed the NetCDF files into raster files using *terra* package. We extracted the values of each variable from these rasters for the Benin Republic and organized them as a data frame stored in an MS Excel sheet.

### Generating data

The downloaded data were not used directly. Only the mean values of each variable and the linear correlation coefficient (*r*) among them were extracted from the database. The mean values were used to simulate many randomly distributed data with three sample sizes (100, 300, and 500). The coefficients *r* were used to simulate the covariance matrix, which introduces the correlation rate in the datasets. Different datasets of different numbers of predictors (5, 8, and 12) were simulated. Three levels of *r* were considered:

- Low correlation rate: |r|≤0.3.
- Moderate correlation rate: 0.3≤|r|≤0.7.
- Migh correlation rate: 0.7≤|r|≤1.

The simulation was made using the *mvrnorm* function from the MASS package in R.

Our purpose being to evaluate the performance of ML in explaining the relationship between the response variable (*y*) and predictors (the matrix *X*), we used the non-linear Eq (1). This formulation aims to challenge ML algorithms with diverse functional relationships (linear, non-linear, and interactive), ensuring a robust evaluation of their predictive capabilities. To simulate *y*, we designed a composite non-linear function inspired by [49], that incorporates a range of common patterns observed in real-world data. Specifically, the function includes (i) an additive quadratic terms $\left[X_{ij}\left(1+X_{ij}\right)=X_{ij}+X_{ij}^{2}\right]$ to model non-linear effects of individual predictors, (ii) an inverse exponential product term to reflect complex multiplicative interactions ($\frac{1}{1+\Pi e^{x_{ij}}}$), and (iii) a logarithmic term $\left[\log\left(\sum X_{ij}\right)\right]$ to simulate the cumulative contribution of all predictors, while controlling the effect of extreme values. For j∈{1,2,...,n},


$$\begin{array}{c} y_{j}=\sum_{i=1}^{p}X_{ij}(1+X_{ij})+\frac{1}{1+\prod_{i=1}^{p}e^{X_{ij}}}+\log(\sum_{i=1}^{p}X_{ij}). \end{array}$$ (1)

### Introducing imperfection

Previously, we generated datasets with different collinearity rates between the predictors. We had *low*, *moderate*, and *high* correlation rates mimicking the multicollinearity in any real datasets. That was the first imperfection. In addition, we introduced another imperfection, which is the missing values. It consists of randomly removing some lines from the initial dataset. We considered four levels of missing rate: 5%, 10%, 20%, and 25%. Missing values were introduced considering the technique of Missing Completely At Random (MCAR). This was operationalized by randomly selecting data points across the predictor matrix and setting them to NA. This choice was guided by our goal of evaluating the robustness of machine learning algorithms and imputation methods under purely random data loss.

### Pre-processing methods studied

Pre-processing a dataset is to improve its quality to meet the desired information when collecting data. One of the common pre-processing methods in statistical analysis is the imputation of missing values. Four common and mostly used imputation techniques were considered: replacement by mean or median values, and regression-based imputation using the lasso model and random forest (R-mice package). The latest methods are multiple imputation by chained equations (MICE), aiming at imputing missing values in an incomplete column (the target variable) by generating plausible synthetic values based on the information from other variables in the dataset [50,51]. Each method was tested under equal missingness assumptions. Mean and Median imputation were selected for their status as the most widely used univariate baseline methods in agricultural prediction tasks, providing a necessary reference point against which more sophisticated techniques can be meaningfully evaluated. MICE-based imputations were selected because they explicitly account for the multivariate covariance structure of the data through a chained equations framework: a valuable property in agricultural datasets where missing values frequently occur across structurally related predictors, such as meteorological or remote sensing variables measured from the same source.

Another pre-processing is the use of principal components (PCs) of the initial (or original) dataset. For that, we analysed the PCs of each dataset and derived the first 2, 3, and 5 PCs, and also considered the initial dataset for comparison, making four levels. The number of retained PCs (2, 3, and 5) was chosen based on the cumulative proportion of explained variance, following a common heuristic in PCA-based pre-processing. These levels generally captured a substantial amount of variability in the data while reducing dimensionality.

### Summary of the scenarios

As summarized in Table 1, for each ML algorithm, we ran 1,728 combinations of factors. We explored 03 levels of number of predictor variables (5, 8, and 12), 3 levels of sample size (100, 300, and 500), 4 levels of missing value rate (5%, 10%, 20%, and 25%), 3 levels of multicollinearity (low, medium, and high), and 4 levels of imputation methods (mean, median, Lasso-regression, and RF-regression).

**Table 1:** Studied factors and their levels.

Variable	Notation	Levels	Number of levels
Number of predictors	n.variables	5, 8, and 12	3
Sample size	samp.size	100, 300, and 500	3
Missing rate	missing.rate	5%, 10%, 20%, and 25%	4
Collinearity rates	cor.rate	low, medium, and high	3
Imputation methods	imputation	mean, median, Mice-Lasso, Mice-RF	4
Principal components	pr.comp	initial, 2, 3, and 5	4
Total number of scenarios	–	–	1,728 (3 × 3 × 4 × 3 × 4 × 4)

### Machine learning modeling and performance evaluation

To achieve our goal, we applied seven of the commonly used ML algorithms in CYP studies. Random Forest (RF) [52,53], Support Vector Regression (SVM) with kernel “radial” [54,55], Multiple Linear Regression (MLR) with ordinary least square estimation method [56,57], and k-Nearest Neighbor (kNN) [58,59] were explored using R-caret package, wereas Extreme Gradient Boosting (XGBoost) and Light Gradient Boosting Machine (LGBM) were explored using R-XGBoost package. Hyperparameter optimization was performed using an automated grid search procedure within the caret package in R (Table 2). This procedure systematically explored the parameter space for each learner, selecting the specific configuration that optimized the *R*^2^ and *MAE* for each simulated scenario and practical crop case. This automated tuning approach was implemented to maintain consistency across the diverse data configurations and to prevent subjective bias in model selection. To ensure model robustness and prevent overfitting, the optimal configuration was selected based on the lowest Mean Squared Error (MSE) derived from 5-fold cross-validation. Each of the seven ML algorithms was applied on the 1,728 scenarios. Hence, we ran in total 12,096 scenarios. All the simulations were run under the R programming language version 4.5.1 with an HP Intel(R), Core(TM) i5-8350U, CPU 1.70GHz (1.90 GHz), and RAM 16 GB.

**Table 2:** Summary of hyperparameter grids used in grid search across all ML algorithms.

Algorithm	Hyperparameter	Values Explored	Selection Criterion
**RF (Random Forest)**	mtry	2, $\lfloor \sqrt{p}\rfloor$, *p*	Grid search — min. MAE
	splitrule	variance	Fixed
	min.node.size	1, 5	Grid search — min. MAE
	num.trees	470	Fixed
**SVM**	Kernel	Radial Basis Function (RBF)	Fixed
	sigma (γ)	0.1	Fixed
	C	1, 4	Grid search — min. MAE
**kNN**	k	1, 3, 5, 7, 9, ..., min(20, n−1)	Grid search — min. MAE
**XGBoost**	objective	reg:squarederror	Fixed
	max_depth	3, 6	Grid search — min. CV-RMSE
	eta (learning rate)	0.05, 0.1	Grid search — min. CV-RMSE
	subsample	0.8, 1.0	Grid search — min. CV-RMSE
	nrounds	1–100 (early stop, patience = 10)	Grid search — min. CV-RMSE
**LightGBM**	objective	regression	Fixed
	metric	rmse	Fixed
	learning_rate	0.1	Fixed
	num_iterations	50	Fixed
	feature_pre_filter	FALSE	Fixed
**NNet**	size (hidden units)	3, 5	Grid search — min. MAE
	decay (L2 penalty)	0.1	Fixed
**MLR**	Estimation method	Ordinary Least Squares (OLS)	Fixed — no tuning required

*Note:* RF  =  Random Forest; SVM  =  Support Vector Machine; p   =   Number of predictors; kNN  =  k-Nearest Neighbours; NNet  =  Neural Network; MLR  =  Multiple Linear Regression. CV  =  cross-validation; MAE  =  Mean Absolute Error; RMSE  =  Root Mean Squared Error. Grid search selects the configuration minimising MAE (or CV-RMSE for XGBoost) on the training set.

To evaluate model performance, we employed a repeated random sub-sampling validation approach. Each simulated dataset was split into two sets, one for training (70%) and the other for testing (30%). This procedure was iterated five times. The coefficient of determination (*R*^2^) and the mean absolute error (MAE) were used as the performance metrics. *R*^2^ values indicate the fit quality, and MAE the error magnitude.


$$\begin{array}{c} MAE=\frac{1}{n}\sum_{i=1}^{n}\left|y_{i}-{\hat{y}}_{i}\right| \end{array}$$ (2)

$$\begin{array}{c} R^{2}=1-\frac{\sum_{i=1}^{n}{\left(y_{i}-{\hat{y}}_{i}\right)}^{2}}{\sum_{i=1}^{n}{\left(y_{i}-\overset{―}{y_{i}}\right)}^{2}} \end{array}$$ (3)

In addition to *R*^2^ and MAE, two complementary metrics were computed to broaden the evaluation framework: the Root Mean Squared Error (RMSE) and the Mean Absolute Percentage Error (MAPE) defined as:


$$\begin{array}{c} \text{RMSE}=\sqrt{\frac{1}{n}\sum_{i=1}^{n}(y_{i}-{\hat{y}}_{i}{)}^{2}} \end{array}$$ (4)

$$\begin{array}{c} \text{MAPE}=\frac{1}{n}\sum_{i=1}^{n}\left|\frac{y_{i}-{\hat{y}}_{i}}{y_{i}}\right|\times 100 \end{array}$$ (5)

where $y_{i}$ denotes the observed yield, ${\hat{y}}_{i}$ the predicted yield, and *n* the number of test observations. RMSE was log-transformed (logRMSE) to control the effect of extreme values across the wide range of simulated yield magnitudes, consistently with the log-transformation applied to MAE. MAPE provides an intuitive percentage-based measure of prediction error that facilitates comparison across crops and yield scales. Nevertheless, to ensure a clear and concise results presentation and interpretation, the logRMSE and MAPE results are therefore reported as supplementary information under Table S2 Table to Table S5 Table, which mirror the structure of the main results tables. The conclusions drawn from logRMSE and MAPE are directionally consistent with those obtained from *R*^2^ and logMAE.

### Performance analysis

A multi-way analysis of variance (ANOVA) was performed to assess the effect of the studied factors on the performance of the ML. All the factors cited above (Table 1) were considered as fixed. We adjusted the interactions so that the residuals match the normal distribution as closely as possible. After adjusting the model, we obtained the trend depicted in S1 Fig. Although the residual histograms appear approximately symmetric and centered around zero, the Q–Q plots exhibit systematic deviations from the theoretical normal distribution, particularly in the tails. These deviations indicate the presence of heavy-tailed error behavior, inconsistent with the Gaussian error assumption required for reliable inference in parametric models. Furthermore, residuals versus fitted value plots reveal non-constant variance, with increasing dispersion at higher fitted values, suggesting heteroscedasticity. Such patterns violate the homoscedasticity assumption and imply that error variance is not independent of the predicted response. Importantly, these deviations are not marginal artefacts but are observed consistently across both performance metrics (R² and MAE), indicating that the error structure is intrinsically non-Gaussian and heteroscedastic, rather than the result of random sampling variation. Hence, we pass to permutation ANOVA, a non-parametric test ran with R−vegan and R−rstatix packages, followed by the Tukey post hoc test with R−emmeans. Significant factors were visualized via boxplots, with the MAE log-transformed to control the effect of extreme values and facilitate the interpretations. logMAE preserves the ordinal structure of the original metric (a lower logMAE still unambiguously indicates better predictive accuracy) and all conclusions drawn from logMAE are directionally consistent with those that would be obtained from the untransformed MAE.

### Practical implementation

To join practice with theory, we considered the data of some of the most widely consumed crops in Benin to predict their yield according to the theories generated through this study. For each of these crops (maize, sorghum, yams, and peanuts), we selected four of the highest-producing municipalities and collected yield data through the DSA platform (https://dsa.agriculture.gouv.bj/), gathering Benin’s crop yield data. Corresponding weather data (temperature, rainfall, water vapour pressure, evapotranspiration, wind speed, and relative humidity) were collected as described in the section Original data acquisition and pre-processing. Importantly, these datasets involved different characteristics (Table 3). The missingness level of each dataset was classified according to the following thresholds: No missingness (0%), Low ([0%, 7%[), Moderate ([7%, 15%[), and High (≥15%).

**Table 3:** Characteristics of the real datasets used for practice.

Data characteristics	Maize	Sorghum	Yam	Peanuts	Cassava
Sample size	104	104	104	104	104
Number of predictors	6	6	6	6	6
Avg Multicollinearity (|*r*|)	0.453	0.347	0.285	0.638	0.456
Rows with missing values	9 (8.65%)	6 (5.77%)	9 (8.65%)	0 (0.00%)	4 (3.85%)

## Results

These results are based on the significance of the main effect of the studied factors (i.e., number of predictor variables, sample size, missing value rate, multicollinearity, and imputation methods) and the interaction terms from the ANOVA. Table 4 shows that each of the factors significantly affects the performance metrics. In addition, in most cases, the interaction between missingness and imputation method was statistically significant (p−value<0.05), indicating that the effect of missing data depends strongly on how it is handled. For simplicity, we present only 12 interactions in addition to the main effects of the 07 factors considered.

**Table 4:** Full Analysis of Variance (ANOVA) results for *R*^2^ and MAE including Effect Sizes.

Source of Variation	*R* ^2^			MAE		
	F	Pr(>F)	$\eta_{p}^{2}$	F	Pr(>F)	$\eta_{p}^{2}$
samp.size	379.18	<0.001***	0.2300	121.63	<0.001***	0.2500
n.variables	18.34	<0.001***	0.0100	98.63	<0.001***	0.6900
imputation	2929.07	<0.001***	0.6600	5.83	0.010**	0.0500
Algorithm	285.52	<0.001***	0.2600	98.59	<0.001***	0.0300
pr.comp	100.06	<0.001***	0.1100	2.28	0.007**	0.0500
cor.rate	300.76	<0.001***	0.2500	39.38	<0.001***	0.2300
missing.rate	52.18	<0.001***	0.0600	75.07	<0.001***	0.0700
Algorithm:n.variable	2.21	0.039*	0.0044	2.16	0.044*	0.0035
Algorithm:missing.rate	2.81	<0.001***	0.0101	0.66	<0.001***	0.0021
Algorithm:imputation	482.68	<0.001***	0.5002	2.66	0.006**	0.1904
Algorithm:samp.size	31.38	<0.001***	0.0703	1.98	0.037*	0.0102
Algorithm:pr.comp	20.73	<0.001***	0.0701	2.73	<0.001***	0.0205
pr.comp:cor.rate	23.05	<0.001***	0.0504	2.79	0.010*	0.0201
imputation:pr.comp	29.57	<0.001***	0.0703	1.98	0.037*	0.0094
imputation:cor.rate	87.17	<0.001***	0.1205	7.87	<0.001***	0.0304
pr.comp:n.variables	3.15	0.020*	0.0071	2.23	0.037*	0.0051
imputation:missing.rate	3.95	0.001**	0.0102	10.11	<0.001***	0.0092
missing.rate:cor.rate	3.90	0.001**	0.0101	64.36	<0.001***	0.0203
n.variables:samp.size	11.67	0.001**	0.0022	76.00	<0.001***	0.1105

Note: Significance levels: *p < 0.05, **p < 0.01, ***p < 0.001. $\eta_{p}^{2}$ = Partial Eta Squared (effect size). samp.size = sample size; n.variables = number of predictors; pr.comp = number of principal components; cor.rate = correlation rate; imputation = imputation methods.

The Tukey post hoc test indicated that Random Forest (RF) achieved significantly higher predictive accuracy (*R*^2^ = 0.80, Group: a) than all other evaluated algorithms (Table 5). While SVM ranked second in *R*^2^ (*R*^2^ = 0.75), it was statistically equivalent to RF in terms of minimizing absolute error (Group: A). Conversely, MLR demonstrated significantly lower performance across all metrics, occupying the lowest significance groups. Regarding data preparation, Mice-based imputation methods (RF and Lasso) formed a superior significance group (Group: a for *R*^2^; Group: A for MAE), significantly outperforming simple Mean and Median imputation, which showed no significant differences between themselves.

**Table 5:** Estimated marginal means and significance groupings for Algorithms and Imputation methods.

	*R* ^2^				MAE			
**Algorithms**								
**Levels**	**EMM**	**LCI**	**UCI**	**Group**	**EMM**	**LCI**	**Upper Cl**	**Group**
MLR	0.0742	0.0673	0.0811	f	3.5	3.47	3.53	E
LGBM	0.4682	0.4613	0.4751	e	2.88	2.86	2.91	C
NNet	0.6144	0.6075	0.6213	d	2.73	2.71	2.76	B
XGBoost	0.6155	0.6086	0.6224	d	3.23	3.2	3.26	D
KNN	0.6326	0.6257	0.6395	c	2.73	2.71	2.76	B
SVM	0.7558	0.7489	0.7627	b	2.37	2.34	2.39	A
RF	0.8008	0.7939	0.8076	a	2.33	2.3	2.35	A
**Imputation method**								
**Levels**	**EMM**	**LCI**	**UCI**	**Group**	**EMM**	**LCI**	**UCI**	**Group**
Mean	0.551	0.546	0.556	b	2.87	2.85	2.89	B
Median	0.552	0.547	0.557	b	2.87	2.85	2.89	B
Mice Lasso	0.578	0.573	0.583	a	2.78	2.76	2.8	A
Mice RF	0.583	0.578	0.588	a	2.79	2.77	2.81	A

EMM = Estimated marginal means; LCI = Lower bound confidence interval; UCI = Upper bound confidence interval.

The graphical trend is clearly depicted in S2 Fig and S3 Fig.

### Performance of ML algorithms

**Fig 2:**
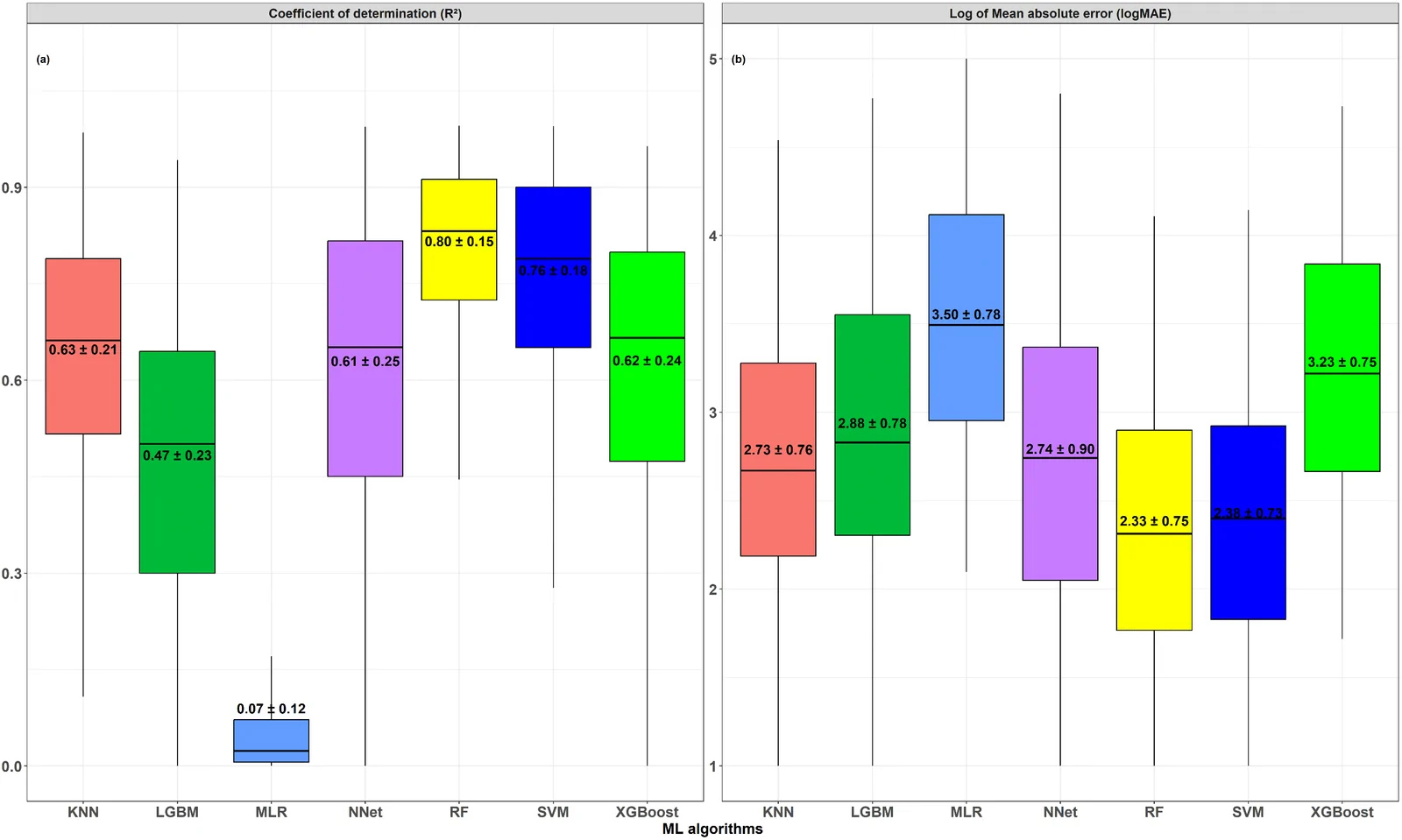
Global performance of learning algorithms.

The predictive performance of the machine learning algorithms varied significantly across the various data structures and quality scenarios (Fig 2). RF emerged as the most robust and accurate model, achieving the highest mean coefficient of determination ($R^{2}=0.80\pm 0.15$) and the lowest error magnitude (logMAE=2.33±0.75). It was closely followed by SVM, which maintained strong stability with an *R*^2^ of 0.76±0.18 and a logMAE of 2.38±0.73. In contrast, gradient boosting algorithms and neural networks showed moderate performance. XGBoost ($R^{2}=0.62\pm 0.24$) and kNN ($R^{2}=0.63\pm 0.21$) exhibited comparable predictive power, while NNet ($R^{2}=0.61\pm 0.25$) and LGBM ($R^{2}=0.47\pm 0.23$) struggled to match the accuracy of the ensemble bagging approach. Notably, the high standard deviations across these models suggest a higher sensitivity to the introduced data imperfections (missingness and multicollinearity) compared to RF and SVM. Finally, MLR demonstrated the poorest performance by a significant margin ($R^{2}=0.07\pm 0.12$; logMAE=3.50±0.78). The near-zero *R*^2^ values indicate that MLR was unable to capture the non-linear dependencies in the crop yield data, particularly when faced with the complex data modalities and imputation-induced noise present in the updated dataset. Overall, RF and SVM proved to be the most reliable algorithms for CYP under varying levels of data quality.

### Analysis of sensitivity

#### Sensitivity of ML to data structure.

The predictive performance of the models was highly sensitive to the dimensions of the training data. As shown in S4 Fig, *R*^2^ values significantly decreased as the number of predictors increased, while the error magnitude increased accordingly. RF consistently outperformed all other algorithms regardless of the number of predictors (Table 6). Nevertheless, with 12 predictors, RF performance decreased to a lower *R*^2^ (0.79 versus 0.81 at 5 predictors) and a higher logMAE (3.11 versus 1.54 at 5 predictors), but still outperformed all competing algorithms, including the second-best SVM (*R*^2^ = 0.75, logMAE = 3.12) and kNN (*R*^2^ = 0.62, logMAE = 3.45). Notably, RF produced the most stable and lowest error rates across 5, 8, and 12 predictors (logMAE of 1.54, 2.39, and 3.11, respectively), systematically below all competing algorithms at every dimensionality level, confirming its superior robustness to high-dimensionality.

**Table 6:** Effect of data structure and imperfection on ML performance (mean ± SD).

Metric	Condition	kNN	LGBM	MLR	NNet	RF	SVM	XGBoost
**Number of Predictors**								
*R* ^2^	5 predictors	0.62±0.25	0.49±0.28	0.13±0.16	0.69±0.26	0.81±0.16	0.76±0.20	0.63±0.25
	8 predictors	0.66±0.18	0.45±0.21	0.03±0.06	0.60±0.24	0.80±0.14	0.75±0.18	0.61±0.23
	12 predictors	0.62±0.18	0.46±0.20	0.05±0.10	0.55±0.24	0.79±0.13	0.75±0.16	0.60±0.23
logMAE	5 predictors	1.96±0.37	2.11±0.44	2.71±0.37	1.84±0.51	1.54±0.36	1.62±0.38	2.45±0.31
	8 predictors	2.79±0.39	2.96±0.45	3.54±0.38	2.82±0.51	2.39±0.38	2.43±0.39	3.30±0.38
	12 predictors	3.45±0.47	3.55±0.44	3.85±0.21	3.51±0.50	3.11±0.45	3.12±0.43	3.77±0.34
**Sample Size**								
*R* ^2^	100 samples	0.54±0.27	0.19±0.15	0.16±0.18	0.54±0.30	0.79±0.17	0.72±0.23	0.58±0.27
	300 samples	0.65±0.18	0.54±0.13	0.05±0.06	0.66±0.23	0.80±0.13	0.77±0.17	0.62±0.23
	500 samples	0.70±0.13	0.64±0.14	0.02±0.03	0.63±0.21	0.82±0.13	0.78±0.14	0.64±0.20
logMAE	100 samples	2.78±0.67	3.11±0.61	3.30±0.58	2.79±0.79	2.37±0.68	2.43±0.66	3.10±0.61
	300 samples	2.76±0.73	2.87±0.72	3.43±0.56	2.68±0.88	2.37±0.77	2.39±0.73	3.23±0.65
	500 samples	2.63±0.73	2.67±0.77	3.34±0.61	2.66±0.87	2.26±0.79	2.31±0.78	3.14±0.66
**Correlation Rate**								
*R* ^2^	Low	0.51±0.19	0.37±0.21	0.07±0.12	0.51±0.25	0.69±0.15	0.50±0.17	0.44±0.23
	Moderate	0.67±0.18	0.50±0.21	0.11±0.15	0.63±0.24	0.82±0.12	0.79±0.13	0.65±0.20
	High	0.71±0.20	0.53±0.25	0.04±0.07	0.70±0.24	0.88±0.09	0.89±0.12	0.74±0.17
logMAE	Low	2.38±0.56	2.47±0.57	2.96±0.55	2.37±0.71	2.08±0.61	2.20±0.62	2.73±0.53
	Moderate	2.78±0.72	2.91±0.72	3.41±0.53	2.80±0.85	2.39±0.74	2.46±0.74	3.24±0.61
	High	2.99±0.77	3.18±0.72	3.68±0.43	2.93±0.88	2.50±0.82	2.40±0.80	3.49±0.54
**Missing Rate**								
*R* ^2^	5%	0.71±0.18	0.50±0.24	0.08±0.14	0.69±0.25	0.88±0.09	0.83±0.15	0.68±0.23
	10%	0.65±0.21	0.48±0.25	0.08±0.14	0.65±0.25	0.88±0.12	0.78±0.16	0.64±0.24
	20%	0.59±0.21	0.43±0.24	0.07±0.11	0.56±0.25	0.76±0.15	0.71±0.19	0.57±0.24
	25%	0.57±0.20	0.47±0.20	0.06±0.09	0.54±0.23	0.72±0.17	0.68±0.20	0.55±0.23
logMAE	5%	2.53±0.77	2.77±0.78	3.35±0.59	2.51±0.94	2.07±0.77	2.11±0.75	3.14±0.66
	10%	2.69±0.74	2.82±0.76	3.35±0.59	2.63±0.88	2.25±0.72	2.31±0.72	3.15±0.65
	20%	2.81±0.72	2.93±0.74	3.36±0.59	2.84±0.79	2.50±0.72	2.54±0.70	3.17±0.65
	25%	2.83±0.65	2.93±0.65	3.35±0.57	2.87±0.70	2.54±0.64	2.58±0.63	3.18±0.60

Sample size also exerted a strong influence on model stability (S5 Fig). *R*^2^ values followed a positive linear trend with increasing sample size (a), while logMAE significantly decreased (b). Analysis of the interactions (Table 6) reveals that RF is particularly dominant in small datasets (100 samples), where it yielded an *R*^2^ of 0.79 compared to the much lower values of XGBoost (0.58) and NNet (0.54). As sample size increased to 500, RF maintained its lead (*R*^2^ = 0.82, logMAE = 2.26), though SVM showed comparable error minimization (logMAE = 2.31). In contrast, MLR performance remained negligible across all sample sizes ($R^{2}\leq 0.16$), and LGBM required larger samples (500) to achieve even moderate accuracy (*R*^2^ = 0.64).

**Fig 3:**
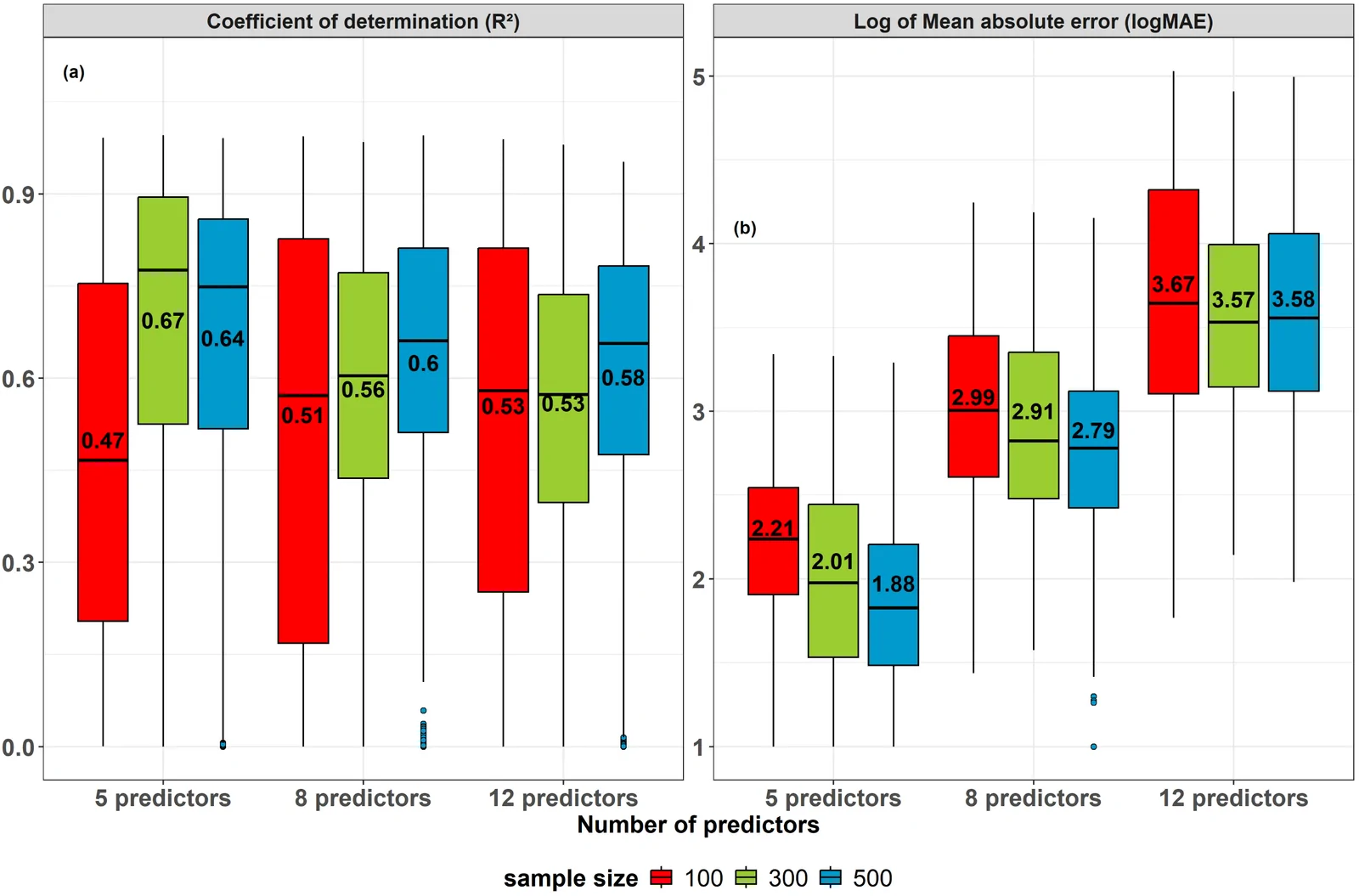
Impact of interaction between sample size and number of predictors on ML performance.

The interaction between sample size and the number of predictors revealed complex patterns in model performance (Fig 3). Generally, *R*^2^ values were highest in datasets with fewer predictors and larger sample sizes, but the stability of this metric was highly dependent on the predictor-to-sample ratio. Specifically, for datasets with 5 predictors, increasing the sample size from 100 to 300 led to a sharp increase in *R*^2^ from 0.47 to 0.67 (Fig 3a). However, as the number of predictors increased to 12, the benefit of a larger sample size on *R*^2^ became less pronounced, with values plateauing between 0.53 and 0.58.

The impact of this interaction was more consistent regarding error magnitude (Fig 3b). Within each predictor modality, increasing the sample size significantly reduced the logMAE. The most favorable performance was observed with 5 predictors and a sample size of 500 (logMAE = 1.88), while the highest error occurred with 12 predictors and only 100 samples (logMAE = 3.67). This indicates that while larger sample sizes can partially mitigate the noise introduced by high-dimensional data, the number of predictors remains a dominant factor in determining the absolute error of the CYP.

Across these interactions, RF demonstrated remarkable resilience (Table 6 and S2 Table). Even in the most challenging scenario (12 predictors and 100 samples), RF maintained a relatively high *R*^2^ of 0.79, whereas the performance of other algorithms, such as LGBM (*R*^2^ = 0.19) and XGBoost (*R*^2^ = 0.58), was substantially more sensitive to the low sample-to-predictor ratio. This suggests that the ensemble bagging approach is more robust to the “curse of dimensionality” within this experimental framework than the boosting or linear alternatives.

#### Sensitivity of ML to data imperfection.

Multicollinearity significantly affected the reliability of the predictions. Both *R*^2^ and MAE increased with the correlation rate, confirming that high multicollinearity leads to model overestimation (S6 Fig). SVM demonstrated this time the highest tolerance to these effects (Table 3), holding an *R*^2^ of 0.89 and the lowest logMAE (2.40) under high correlation conditions. But, it was closely followed by RF (*R*^2^ = 0.88, logMAE = 2.50), suggesting that kernel-based and bagging-based methods are better at navigating redundant predictor spaces than boosting algorithms like XGBoost, which saw error rates rise to 3.49 under high correlation.

The impact of missing data was similarly pronounced (S7 Fig). While an increase in the missing value rate from 5% to 25% led to a general decline in *R*^2^ and an increase in logMAE, the performance gap between algorithms became more apparent. RF achieved the highest *R*^2^ across all missingness levels (0.88 at 5% to 0.72 at 25%).

**Fig 4:**
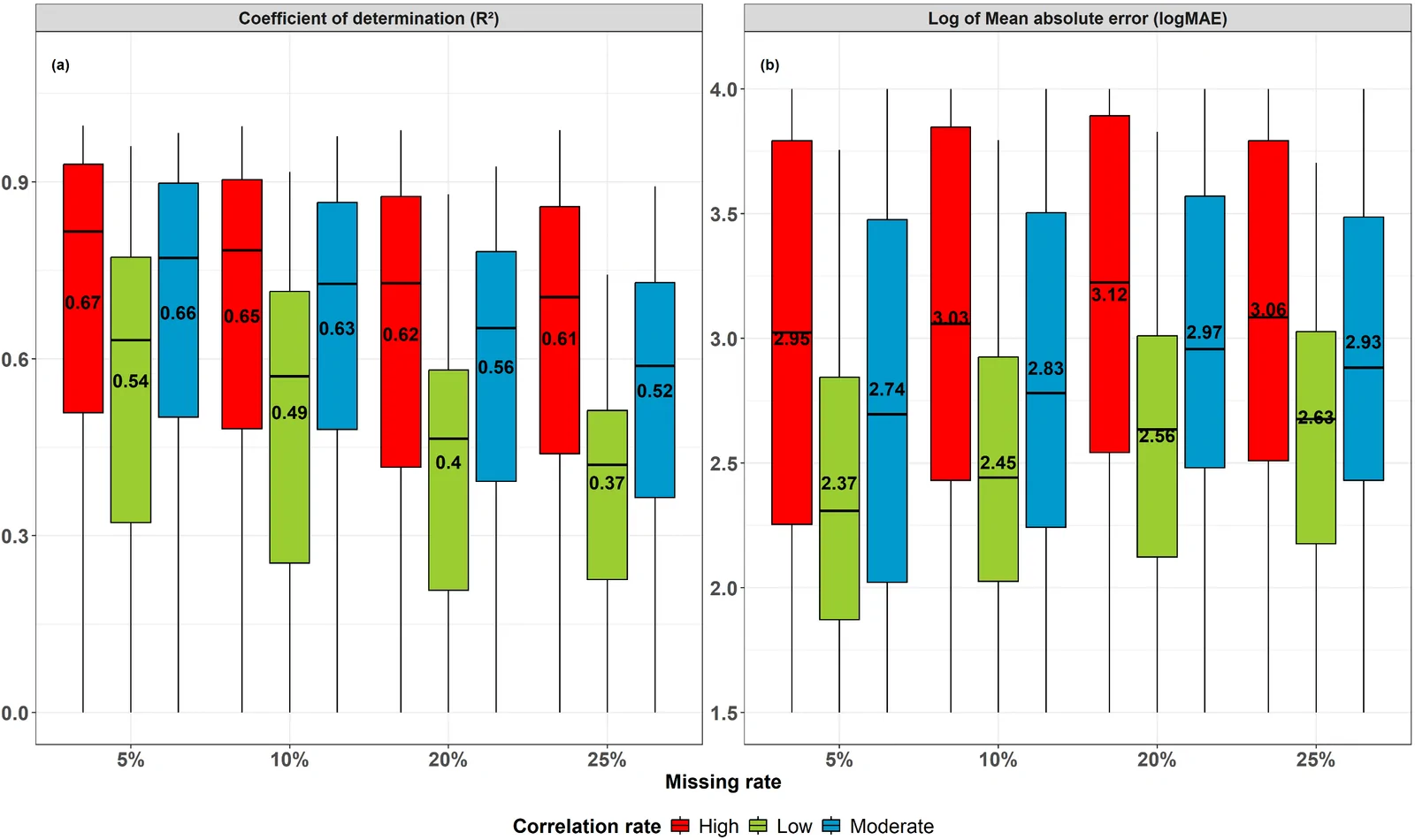
Impact of interaction between missing values and multicollinearity on ML performances.

The interaction between the missing value rate and the correlation rate significantly influenced the predictive stability of the ML models (Fig 4). Across all missingness levels (5% to 25%), datasets with a high correlation rate consistently yielded the highest *R*^2^ values, ranging from 0.67 at a 5% missing rate to 0.61 at a 25% missing rate. In contrast, datasets with low correlation showed the lowest *R*^2^ and the highest sensitivity to missingness (Fig 4a), with performance dropping from 0.54 to 0.37 as the missing rate increased. This suggests that high multicollinearity acts as a structural redundancy that allows models to maintain a high coefficient of determination even when substantial data is missing.

However, this high *R*^2^ in correlated datasets did not translate to higher accuracy. As shown in Fig 4b, the logMAE was consistently highest for the high correlation modality across all missing rates (ranging from 2.95 to 3.06). Conversely, the low correlation modality, despite having the lowest *R*^2^, achieved the best error minimization (logMAE ranging from 2.37 to 2.63).

This interaction highlights a critical phenomenon: high multicollinearity in crop yield data leads to an overestimation of model fit (*R*^2^) while simultaneously increasing prediction error (logMAE). The stability of RF and SVM in these scenarios (Table 3) further indicates that while all models are affected by this interaction, ensemble bagging and kernel methods are more adept at navigating the trade-off between redundancy-driven fit and noise-driven error than linear or boosting-based alternatives.

#### Sensitivity of ML to pre-processing methods.

**Fig 5:**
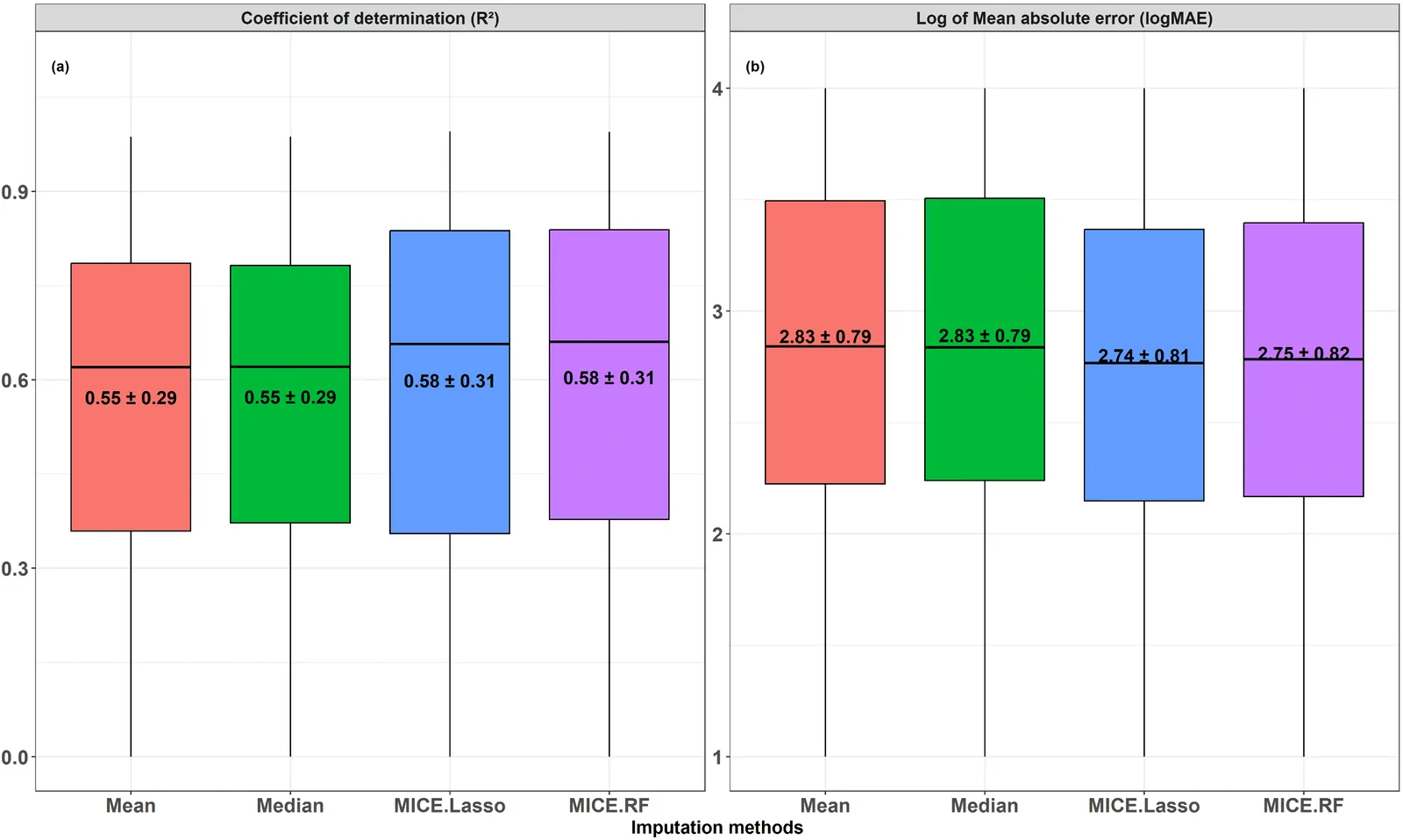
Impact of imputation methods on ML models.

The choice of data pre-processing significantly influenced model performance, with advanced imputation and dimensionality reduction methods yielding the best results. Among the imputation strategies, regression-based methods outperformed simple central tendency measures (Fig 5). Both MICE.Lasso and MICE.RF achieved the highest mean coefficient of determination ($R^{2}=0.58\pm 0.31$), compared to Mean and Median imputation ($R^{2}=0.55\pm 0.29$). Similarly, MICE-based methods were superior error minimizers (Fig 5b), with MICE.Lasso yielding the lowest error (logMAE=2.74±0.81), closely followed by MICE.RF (logMAE=2.75±0.82).

The interaction between imputation methods and specific algorithms further clarifies these trends (Table 7). RF demonstrated high stability across all imputation techniques, achieving its peak performance when paired with MICE.Lasso or MICE.RF ($R^{2}=0.81\pm 0.16$). In contrast, while MLR remained the weakest performer overall, it showed negligible sensitivity to the type of imputation used ($R^{2}\approx 0.08$), suggesting that the structural limitations of linear models in this context cannot be overcome by advanced imputation alone.

**Table 7:** Effect of imputation methods and number of principal components across ML algorithms (mean ± SD).

Metric	Condition	kNN	LGBM	MLR	NNet	RF	SVM	XGBoost
**Imputation Method**								
*R* ^2^	Mean	0.61±0.20	0.46±0.23	0.08±0.12	0.60±0.24	0.80±0.13	0.74±0.18	0.58±0.23
	Median	0.61±0.20	0.46±0.23	0.08±0.13	0.59±0.25	0.79±0.13	0.74±0.16	0.60±0.23
	MiceLasso	0.65±0.22	0.47±0.24	0.07±0.12	0.64±0.26	0.81±0.16	0.77±0.20	0.63±0.25
	RegressionRF	0.66±0.20	0.48±0.24	0.07±0.12	0.64±0.26	0.81±0.16	0.77±0.19	0.65±0.24
logMAE	Mean	2.78±0.76	2.91±0.78	3.50±0.78	2.86±0.88	2.39±0.73	2.47±0.73	3.23±0.75
	Median	2.78±0.76	2.91±0.78	3.50±0.78	2.80±0.88	2.39±0.76	2.47±0.72	3.24±0.75
	MiceLasso	2.70±0.73	2.86±0.76	3.50±0.78	2.65±0.92	2.26±0.27	2.41±0.71	3.23±0.74
	RegressionRF	2.68±0.76	2.85±0.79	3.50±0.78	2.68±0.92	2.29±0.07	2.36±0.73	3.23±0.75
**Number of PCs**								
*R* ^2^	2 PCs	0.69±0.18	0.47±0.22	0.08±0.13	0.71±0.20	0.84±0.12	0.77±0.17	0.69±0.19
	3 PCs	0.61±0.19	0.46±0.23	0.08±0.14	0.67±0.24	0.82±0.13	0.77±0.17	0.68±0.19
	5 PCs	0.52±0.20	0.45±0.24	0.07±0.11	0.56±0.25	0.78±0.14	0.77±0.16	0.67±0.19
	Initial	0.71±0.20	0.49±0.25	0.06±0.10	0.51±0.26	0.75±0.17	0.72±0.22	0.42±0.26
logMAE	2 PCs	2.58±0.72	2.84±0.79	3.49±0.78	2.51±0.83	2.21±0.70	2.28±0.71	3.20±0.75
	3 PCs	2.78±0.74	2.86±0.79	3.50±0.77	2.64±0.81	2.29±0.03	2.70±0.71	3.20±0.75
	5 PCs	2.99±0.76	2.88±0.80	3.50±0.78	2.90±0.92	2.46±0.74	2.31±0.72	3.21±0.75
	Initial	2.59±0.72	2.95±0.73	3.51±0.78	2.94±0.96	2.37±0.82	2.62±0.73	3.31±0.74

**Fig 6:**
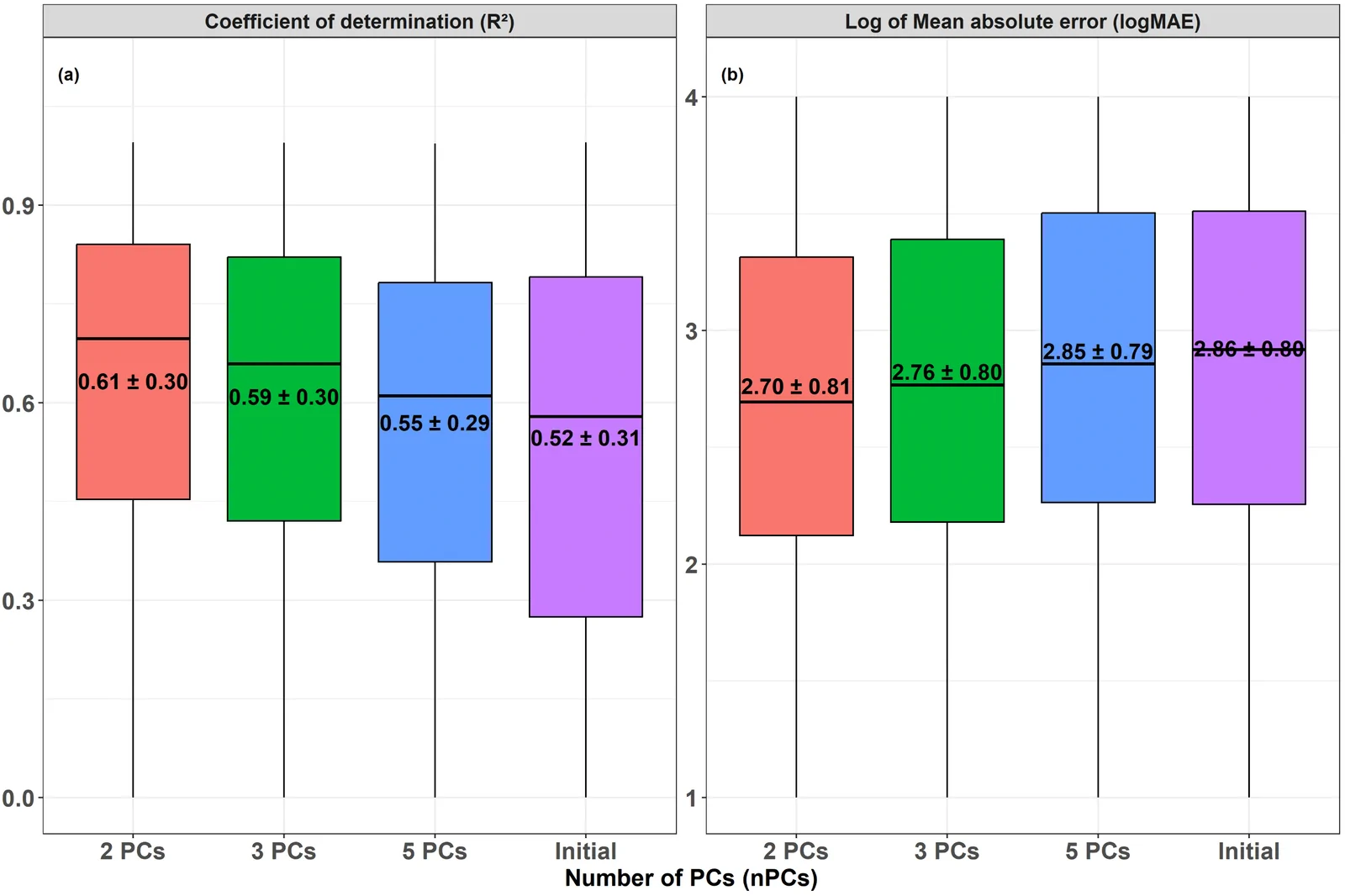
Impact of principal components on ML models.

Regarding dimensionality reduction, the results indicate that a lower number of principal components (PCs) can enhance model fit and reduce noise (Fig 6). The 2 PCs modality achieved the highest perforance ($R^{2}=0.61\pm 0.30$; logMAE=2.70±0.81 ), significantly outperforming the Initial dataset ($R^{2}=0.52\pm 0.31$; logMAE=2.86±0.80). Performance consistently degraded as the number of PCs increased from 2 to 5, where *R*^2^ dropped to 0.55 and logMAE rose to 2.85.

The combination of algorithm and PCA modality revealed that ensemble and kernel-based methods benefit most from feature extraction (Table 7 and S3 Table). The RF + 2 PCs combination emerged as the optimal configuration, yielding the highest *R*^2^ (0.84) and the lowest logMAE (2.21). Similarly, SVM and NNet showed improved error minimization with 2 PCs (logMAE of 2.28 and 2.51, respectively) compared to their performance on the initial datasets. These results suggest that for CYP under data imperfection, reducing the predictor space to 2 PCs effectively filters out noise while retaining the essential structural information required for high-accuracy modeling.

#### Other interactions between data structure, imperfection, and pre-processing.

The interaction between data quality factors and dimensionality reduction significantly influenced predictive outcomes (Table 8 and S4 Table). Regardless of the dataset type (Initial or PCs), an increase in the correlation rate led to a higher coefficient of determination (*R*^2^) but was accompanied by an increase in error magnitude (logMAE). The 2 PCs configuration consistently yielded the superior performance across all multicollinearity levels, achieving the highest *R*^2^ for low (0.52±0.26), moderate (0.64±0.29), and high (0.66±0.32) correlation rates. Notably, under high multicollinearity, 2 PCs offered better error minimization (logMAE=2.98±0.81) than the initial dataset (logMAE=3.01±0.84), suggesting that dimensionality reduction effectively filters out redundancy-induced noise.

**Table 8:** Effect of interaction between multicollinearity or number of predictors and principal components (mean ± SD).

Metric	Condition	2 PCs	3 PCs	5 PCs	Initial
**Multicollinearity**					
*R* ^2^	Low	0.52±0.26	0.49±0.26	0.46±0.25	0.35±0.25
	Moderate	0.64±0.29	0.61±0.29	0.57±0.28	0.56±0.28
	High	0.66±0.32	0.65±0.32	0.60±0.32	0.65±0.32
logMAE	Low	2.40±0.59	2.44±0.59	2.49±0.58	2.63±0.64
	Moderate	2.76±0.76	2.83±0.75	2.92±0.75	2.95±0.76
	High	2.98±0.81	3.03±0.80	3.14±0.79	3.01±0.84
**Number of predictors**					
*R* ^2^	5 predictors	0.62±0.30	0.60±0.31	0.57±0.31	0.58±0.32
	8 predictors	0.60±0.30	0.57±0.30	0.54±0.29	0.52±0.31
	12 predictors	0.61±0.29	0.58±0.28	0.53±0.28	0.47±0.30
logMAE	5 predictors	1.98±0.56	2.02±0.56	2.10±0.54	2.04±0.55
	8 predictors	2.79±0.61	2.86±0.58	2.95±0.60	2.97±0.50
	12 predictors	3.36±0.54	3.42±0.52	3.52±0.50	3.61±0.36

A similar trend was observed in the interaction between the number of predictors and principal components. As the predictor count increased from 5 to 12, there was a general decline in model performance across all modalities. However, the 2 PCs transformation proved highly effective at mitigating the “curse of dimensionality.” For datasets with 12 predictors, the 2 PCs modality achieved an *R*^2^ of 0.61±0.29 and a logMAE of 3.36±0.54, whereas the initial dataset performed significantly worse with an *R*^2^ of 0.47±0.30 and a logMAE of 3.61±0.36.

Interestingly, when the number of predictors was low (5 predictors), the performance gap between the 2 PCs modality ($R^{2}=0.62\pm 0.30$, logMAE=1.98±0.56) and the initial dataset ($R^{2}=0.58\pm 0.32$, logMAE=2.04±0.55) was less pronounced. This indicates that while dimensionality reduction via 2 PCs is beneficial across all scenarios, its impact on enhancing predictive accuracy and error minimization is most critical when dealing with high-dimensional and highly correlated crop yield datasets.

The interaction between imputation methods and structural data modifications (dimensionality reduction and missingness) revealed distinct performance patterns (Table 9 and S5 Table). Regarding the interaction with principal components, advanced imputation methods exhibited superior error minimization across all PC modalities. Specifically, for the 2 PCs dataset, Mice-Lasso and Regression-RF achieved the lowest error (logMAE=2.67±0.77 and 2.68±0.78, respectively) compared to Mean and Median imputation (logMAE≈2.77). While *R*^2^ values remained relatively stable across imputation types within each PC level, the 2 PCs modality consistently outperformed the Initial dataset and higher PC counts, reaching an *R*^2^ of 0.62±0.31 when paired with advanced imputation.

**Table 9:** Effect of interaction between number of principal components or missing rate and imputation methods (mean ± SD).

Metric	Condition	Mean	Median	MiceLasso	RegressionRF
**Number of PCs**					
*R* ^2^	2 PCs	0.60±0.29	0.60±0.29	0.62±0.31	0.62±0.31
	3 PCs	0.57±0.29	0.57±0.29	0.60±0.31	0.60±0.31
	5 PCs	0.54±0.28	0.53±0.29	0.56±0.31	0.56±0.30
	Initial	0.50±0.31	0.50±0.30	0.54±0.32	0.55±0.31
logMAE	2 PCs	2.77±0.76	2.76±0.76	2.67±0.77	2.68±0.78
	3 PCs	2.82±0.75	2.82±0.75	2.73±0.76	2.74±0.77
	5 PCs	2.88±0.76	2.89±0.76	2.82±0.76	2.84±0.77
	Initial	2.90±0.77	2.92±0.76	2.83±0.77	2.83±0.78
**Missing Rate**					
*R* ^2^	5%	0.62±0.31	0.62±0.31	0.65±0.31	0.66±0.31
	10%	0.60±0.31	0.60±0.31	0.63±0.31	0.64±0.31
	20%	0.56±0.31	0.56±0.30	0.59±0.30	0.60±0.30
	25%	0.54±0.32	0.53±0.32	0.56±0.31	0.57±0.31
logMAE	5%	2.74±0.77	2.75±0.77	2.64±0.78	2.65±0.79
	10%	2.84±0.77	2.84±0.77	2.75±0.77	2.76±0.77
	20%	2.91±0.77	2.91±0.77	2.83±0.77	2.84±0.77
	25%	2.92±0.76	2.93±0.76	2.85±0.76	2.86±0.76

The impact of the missing value rate on imputation effectiveness was equally pronounced. As missingness increased from 5% to 25%, the predictive power declined across all scenarios; however, advanced techniques maintained a clear advantage. At a 25% missing rate, MiceLasso and RegressionRF yielded higher *R*^2^ values (0.56±0.31 and 0.57±0.31, respectively) compared to the Mean and Median methods (0.54±0.32 and 0.53±0.32).

Furthermore, the error magnitude was consistently lower for regression-based imputations at every level of missingness. For instance, at 5% missingness, MiceLasso achieved a logMAE of 2.64±0.78, while Mean and Median imputation resulted in higher errors (2.74±0.77 and 2.75±0.77, respectively). This trend persisted up to the 25% missing rate, where Mice.Lasso (logMAE=2.85±0.76) continued to outperform simple central tendency imputations (logMAE≈2.93). These findings suggest that MiceLasso and RegressionRF are more resilient to high rates of data missingness and integrate more effectively with dimensionality reduction techniques to preserve the predictive integrity of crop yield models.

#### Optimal actions on the common challenges faced in imperfect datasets pre-processing for ML-based prediction.

This study evaluated the predictive performance of two-way interactions. The comprehensive analysis reveals a clear hierarchy of factors governing predictive performance in crop yield modeling. The results indicate that the robustness of any given algorithm is intrinsically tied to the specific pre-processing interventions applied to address data structural and quality challenges.

**Small-sample and high dimensionality:** the interaction between sample size and the number of predictors confirms that while performance generally improves with larger samples and lower dimensionality, Random Forest (RF) maintains a unique lead in small-sample (*N* = 100) and high-dimensional (12 predictors) scenarios. While newer boosting algorithms such as LGBM and XGBoost require larger data volumes to achieve stability, RF and SVM demonstrate superior resilience across varying predictor-to-sample ratios.

**Multicollinearity and missingness:** regarding data imperfections, the interaction between multicollinearity and missingness rates shows that high correlation creates a deceptive increase in *R*^2^ while simultaneously inflating the logMAE. This “overestimation effect” is most effectively mitigated through the extraction of 2 PCs, which filters redundant noise and stabilizes the error magnitude regardless of the missingness level.

**Pre-processing:** Furthermore, the interaction between imputation methods and algorithmic performance reveals that advanced MICE-based regression (Lasso and RF) consistently outperforms simple central tendency measures. These advanced techniques restore the underlying data distribution more accurately, allowing ensemble models to achieve peak accuracy even at a 25% missing rate. Finally, the interaction between dimensionality reduction and machine learning algorithms highlights a “less is more” trend; using 2 PCs consistently yields better error minimization and higher *R*^2^ than the initial dataset or higher PC counts by providing a cleaner, non-redundant feature space for RF and SVM.

In summary, for imperfect agricultural datasets, the most reliable workflow involves the use of MICE-regression paired with 2 PCs extraction. Under these optimized conditions, Random Forest emerges as the most powerful and robust algorithm, although SVM and kNN serve as effective alternatives when the predictor space is properly reduced and missing information is intelligently restored.

#### Validation through practical crop datasets.

The empirical validation across five distinct crop datasets (Sorghum, Yam, Maize, Peanuts, and Cassava) confirms that predictive success in agriculture is a function of the synergy between data characteristics and the modeling pipeline (Table 10). RF emerged as the top-performing algorithm for three of the five crops: Sorghum (*R*^2^ = 0.58), Yam (*R*^2^ = 0.51), and Cassava (*R*^2^ = 0.22), reinforcing its simulation-established robustness to noise and non-linearities inherent in Benin field data. In datasets with moderate missingness and low collinearity, such as Yam and Sorghum, MICE-based imputation methods served as critical stabilizers, allowing the models to capture underlying yield patterns that simpler imputation strategies would have obscured. For Peanuts, which exhibited no missingness but high collinearity, applying 2 Principal Components (PCs) without any imputation enabled XGBoost to reach peak performance (*R*^2^ = 0.42), directly validating our simulation finding that dimensionality reduction acts as a vital noise filter when feature redundancy dominates. Interestingly, for Maize (where both moderate missingness and moderate collinearity coexisted), the combination of MICE-RF and 2 PCs restructured the feature space sufficiently for kNN (*R*^2^ = 0.35) to outperform ensemble methods, illustrating that local-pattern learners gain competitive advantage when pre-processing successfully resolves both data imperfections simultaneously.

**Table 10:** Optimal modeling workflows and performance metrics for practical crop yield datasets.

Crop	Data Characteristics	Best Pre-processing	Top Algorithm	R²	logMAE
Sorghum	Low Missingness, Low Collinearity	Mice.RF	RF	0.58	4.655
Yam	Moderate Missingness, Low Collinearity	Mice.Lasso	RF	0.51	7.563
Maize	Moderate Missingness, Moderate Collinearity	Mice.RF + 2 PCs	kNN	0.35	5.398
Peanuts	No Missingness, High Collinearity	2 PCs	XGBoost	0.42	5.31
Cassava	Low Missingness, Moderate Collinearity	Mice.RF + 2 PCs	RF	0.22	8.135

Overall, these results demonstrate that no single algorithm or pre-processing strategy is universally optimal; rather, the strategic alignment between data characteristics, imputation method, and dimensionality reduction consistently determines predictive integrity across diverse agricultural contexts (S6 Table, S7 Table, S8 Table, S9 Table, and S10 Table, respectively). Performance variability should be anticipated when this framework is applied to more diverse datasets characterised by different agro-ecological conditions, crop physiologies, or more severe missingness mechanisms, such as MAR or MNAR patterns where the strategic alignment between data characteristics, imputation method, and dimensionality reduction identified here may yield different optimal configurations, reinforcing that the guidelines proposed in this study should be treated as evidence-based starting points rather than universal prescriptions.

## Discussion

This study demonstrates that machine learning (ML) performance in agricultural yield prediction is a function of data structure, imperfections, and pre-processing. Random Forest (RF) emerged as the most robust algorithm, consistently maintaining high performance across both simulated and practical datasets, regardless of small sample sizes (N≈100) or high-dimensional predictor spaces. While SVM and kNN proved competitive in specific cases, notably with Sorghum (*R*^2^ = 0.58) and Maize (*R*^2^ = 0.35), RF’s stability confirms it as a reliable baseline for complex agricultural data. In predicting potato yield in Spain, [18] highlighted the issue of small sample sizes; despite this, RF provided satisfactory results, which aligns with our findings across five distinct crops. Our analysis confirms that identifying the best model is complex because the configuration with the highest *R*^2^ does not always align with the lowest *MAE*. For instance, while SVM yielded a high *R*^2^ for Sorghum, RF provided better error minimization in other contexts. This suggests that relying solely on *R*^2^, as seen in studies like [60], may mask significant predictive errors. We therefore recommend a multi-metric evaluation, as our ANOVA results showed that the interaction between Algorithm and Imputation had a massive effect size ($\eta_{p}^{2}=0.50$ for *R*^2^), indicating that model selection cannot be decoupled from data remediation strategy.

Data imperfections significantly dictated the effectiveness of pre-processing. In crops with high multicollinearity, like Peanuts (|*r*| = 0.63), dimensionality reduction through 2 PCs was essential to stabilize algorithms like XGBoost. This aligns with the findings of [61], who noted that multicollinearity is a primary limitation in regression-based models. The “multicollinearity paradox” observed in our results (where *R*^2^ increases while *MAE* worsens) is fundamentally a byproduct of overfitting. In high-collinearity environments, the model overestimates its predictive power by fitting to the redundant overlap between predictors. This leads to a mathematical over-adjustment to training noise, which manifests as an inflated *R*^2^ but results in large deviations in out-of-sample predictions (MAE). The multicollinearity paradox arises through two complementary mechanisms. The first is a **coefficient instability mechanism**: under high collinearity, the near-singularity of the predictor matrix destabilises model parameters, producing erratic point-level predictions that inflate MAE even when the global fit appears acceptable. The second is a **variance inflation mechanism**: when predictors are highly correlated, their joint contribution to the response variable amplifies the total variance of Y (SS_*T*_), which causes R${\;}^{2}=1-{\text{SS}}_{E}/{\text{SS}}_{T}$ to increase mechanically as the denominator grows, even when residuals also grow. While this second mechanism is embedded in our simulation design, it is not an artefact: it reflects a plausible real-world scenario in which correlated agro-climatic predictors jointly amplify yield variability, as is well documented in agricultural systems where temperature, radiation, and soil moisture covary seasonally [62]. Together, these two mechanisms explain why R^2^ and MAE can diverge simultaneously under high multicollinearity, and why R^2^ alone is an unreliable criterion for model selection in agricultural datasets characterised by correlated predictor structures. This supports our finding that PCA acts as a necessary noise filter in correlated environments, consistent with [43], who found that PCs improved accuracy, though their application is not always straightforward.

Conversely, in settings where correlation was managed or low, preserving the original feature space yielded better results, confirming that pre-processing must be tailored to the dataset’s specific structure. The success of advanced imputation (e.g., Mice.RF and Mice.Lasso) in the top configurations for Yam, Maize, and Cassava underscores its superiority over simple mean or median replacement. Our ANOVA results quantified this, showing that Imputation was the most influential factor ($F=2929.07,\eta_{p}^{2}=0.66$), proving that restoring the underlying distribution is more critical than the choice of the learner itself.

From a computational standpoint, Mean and Median imputation are virtually instantaneous, while MICE-based methods carry a higher but one-time burden that scales with sample size and predictor count. Similarly, MLR and kNN are the least demanding algorithms, whereas RF, XGBoost, and LightGBM require ensemble training, with LightGBM mitigating this cost through histogram-based splitting. Practitioners should therefore consider MICE-RF paired with RF as the most reliable workflow when computational resources are available, and Median imputation paired with SVM as a leaner yet competitive alternative.

As limitations of this study, we explored only a subset of algorithms common in yield prediction, while more powerful architectures exist [63,64]. Furthermore, we focused on established ML-based imputation rather than emerging multidimensional meteorological frameworks [65]. Future research should investigate these evolving methods to further refine yield forecasting in data-scarce environments. Interest may also be drawn to quantitative analysis of the trade-off between performance gains and computational expense.

### Limitations and future work

While this study provides a comprehensive and systematic investigation of the sensitivity of ML regression models to data structure and imperfections in crop yield prediction, several limitations should be acknowledged and serve as directions for future research.

Although the simulation framework was carefully designed to reflect realistic agricultural data conditions (including varying sample sizes, predictor counts, collinearity levels, and missing rates) the reliance on simulated data inevitably introduces assumptions that may not fully capture the complexity and heterogeneity of real field environments. The generalisability of the proposed guidelines should therefore be interpreted with this caveat in mind.

In addition, the empirical validation was conducted on five crop datasets (Maize, Yam, Cassava, Sorghum, and Peanuts) collected from a specific agro-ecological context in Benin. While these datasets confirm the practical relevance of our simulation findings, a broader validation encompassing more diverse crops, regions, soil types, and agricultural production systems would further strengthen the external validity of the proposed pre-processing framework.

Furthermore, while seven algorithms covering a broad spectrum from linear (MLR) to kernel-based (SVM), instance-based (kNN), ensemble (RF, XGBoost, LightGBM), and neural (NNet) learners were evaluated, deep learning architectures such as Long Short-Term Memory (LSTM) networks and Convolutional Neural Networks (CNN) were not considered. These architectures may offer additional robustness under specific data conditions, particularly when temporal or spatial structure is present in the predictor variables.

Regarding the evaluation framework, model performance was assessed through R^2^, MAE, logRMSE, and MAPE. Future work should explore complementary uncertainty-aware evaluation frameworks such as quantile regression assessed with pinball loss, which would provide a more comprehensive picture of predictive reliability under imperfect data conditions and better support decision-making under uncertainty in agricultural planning.

Finally, the sensitivity analysis could be further extended by testing model robustness under controlled input perturbations such as Gaussian noise injection into the predictor variables, which would simulate measurement error and sensor drift: common sources of imperfection in field data collection. Hyperparameter optimisation strategies beyond the grid search applied here, such as Bayesian optimisation or random search, may additionally alter algorithm performance rankings under specific data conditions and deserve systematic investigation in future studies.

## Conclusion

This study demonstrated that data structure, imperfections, and pre-processing highly influence ML models. However, RF is the most robust algorithm to data imperfections, confirmed across all conditions examined. Specifically, the practical results for Sorghum and Yam confirm RF and SVM’s stability in small-sample agricultural contexts. Quantitatively, structural factors dictated performance: larger sample sizes improved accuracy (*R*^2^ = +10.81%), while increased dimensionality escalated error (MAE = +47.37%). We identified a “multicollinearity paradox”, where high correlation can inflate *R*^2^ while simultaneously increasing prediction error, as confirmed by the Peanuts dataset (|*r*| = 0.633). Based on these findings, we propose the following prescriptive guidelines for practitioners. When the dataset is small with moderate multicollinearity and a high percentage of missing values, a configuration analogous to the Maize dataset in our validation, we recommend applying MICE-RF imputation to restore data integrity, followed by dimensionality reduction via 2 PCs to neutralise collinearity-induced variance inflation, and fitting a RF model as the primary learner given its demonstrated robustness to noisy and incomplete data. When data are complete but collinearity is high, 2 PCs alone paired with a boosting algorithm such as XGBoost represents the most reliable workflow, as illustrated by the Peanuts results. When missingness is low and collinearity is negligible, preserving the original feature space without imputation or dimensionality reduction, as in the Sorghum workflow, allows SVM or RF to exploit the clean signal most efficiently. Data structure and pre-processing interact significantly, and they should not be treated individually, but must be tailored to the dataset’s specific correlation and missingness profile rather than treated as isolated decisions. Future work should explore more diverse algorithms, evaluation metrics, and missing data mechanisms, including MAR and MNAR patterns, to further generalise these guidelines across broader agricultural contexts.

## Supporting information

S1 Table Bioclimatic variables. Bioclimatic variables used for the simulation.
(XLSX)

S1 Fig Residuals normality check. Distribution of residuals from the parametric ANOVA.
(TIF)

S2 Fig Turkey post hoc test. Estimated marginal mean by Algorithm.
(TIF)

S3 Fig Turkey post hoc test. Estimated marginal mean by Imputation method.
(TIF)

S4 Fig Predictor count impact ML. Impact of the number of predictors on ML performance.
(TIF)

S5 Fig Sample size impacts ML. Impact of sample size on ML performance.
(TIF)

S6 Fig Multicollinearity impacts ML. Impact of multicollinearity on ML performance.
(TIF)

S7 Fig Missingness impacts ML. Impact of missing values on ML performance.
(TIF)

S2 TableTable 6 Continuous.Effect of data structure and imperfection on ML performance (mean ± SD) *Note:* logRMSE = logarithm of Root Mean Squared Error; MAPE = Mean Absolute Percentage Error (%).(XLSX)

S3 TableTable 7 Continuous.Effect of imputation methods and number of principal components across ML algorithms (mean ± SD). *Note:* logRMSE = logarithm of Root Mean Squared Error; MAPE = Mean Absolute Percentage Error (%). MiceLasso = MICE with Lasso; RegressionRF = MICE with Random Forest.(XLSX)

S4 TableTable 8 Continuous.Effect of interaction between multicollinearity or number of predictors and principal components (mean ± SD). *Note:* logRMSE = logarithm of Root Mean Squared Error; MAPE = Mean Absolute Percentage Error (%). Values averaged across all algorithms, imputation methods, sample sizes, and missing rates.(XLSX)

S5 TableTable 9 Continuous.Effect of interaction between number of principal components or missing rate and imputation methods (mean ± SD). *Note:* logRMSE = logarithm of Root Mean Squared Error; MAPE = Mean Absolute Percentage Error (%). MiceLasso = MICE with Lasso; RegressionRF = MICE with Random Forest.(XLSX)

S6 Table Analyzing maize yield. Extended predictive performance for Maize yield across all modeling configurations.
(XLSX)

S7 Table Analyzing yam yield. Extended predictive performance for Yam yield across all modeling configurations.
(XLSX)

S8 Table Analyzing Cassava yield. Extended predictive performance for Cassava yield across all modeling configurations.
(XLSX)

S9 Table Analyzing Sorghum yield. Extended predictive performance for sorghum yield across all modeling configurations.
(XLSX)

S10 Table Analyzing peanuts yield. Extended predictive performance for peanut yield across all modeling configurations.
(XLSX)

S1 Code Simulation materials. The simulation materials are available at https://zenodo.org/records/18607703. The file *run.scenario.R* simulates data using the function *Simulation*_*function*.*R* based on *GData*_*NA*.*xlsx*. It generates the artificial dataset named *simulated*_*data*.*xlsx*, which was analysed with *Analysis*.*R*. The practice section was performed by *Practice*_*SO2*.*R* using the real datasets such as *CassavaKK*.*xlsx*, *GroundnutKK*.*xlsx*, *MaizeKK*.*xlsx*, *SorghumKK.xlsx*, and *YamKK*.*xlsx*.
(ZIP)
