# Supplementary material for: Sensitivity of machine learning regression models to data structure and quality in crop yield prediction
Source: PLoS One. 2026 Sep 1;21(9):e0353938. doi: 10.1371/journal.pone.0353938 (PMC13533432; doi:10.1371/journal.pone.0353938)
Supplement: Supplementary file 1 [file pone.0353938.s021.pdf]

Answers to reviewers' comments on Manuscript No. PONE-D-25-57279

January 29, 2026

Dear Professor Rajib Chowdhury,

On behalf of my co-authors, I would like to thank you for giving us an opportunity to submit a revised version of our manuscript entitled "**Sensitivity of machine learning regression models to data structure and quality in crop yield prediction**" to *PLOS ONE*. I would also like to thank you and the reviewers for the valuable time you devoted to proofreading the manuscript and for your valuable comments.

Your constructive comments have been very helpful in improving the quality and clarity of our manuscript. We provide point-by-point responses to the reviewers' comments below. The comments are reproduced in *italics*, and our responses are provided in regular (upright) font.

Sincerely,

On behalf of Zinzinhédo, Mitchozounnou, Salako, and Glèlè Kakaï.

1 Response to the Academic Editor's comments

1.1 Comment 1:

Please ensure that your manuscript meets PLOS ONE's style requirements, including those for file naming.

Response 1: To the best of our knowledge, the manuscript have been polish to meets PLOS ONE's style requirements.

1.2 Comment 2:

Please note that PLOS One has specific guidelines on code sharing for submissions in which author-generated code underpins the findings in the manuscript. In these cases, we expect all author-generated code to be made available without restrictions upon publication of the work. Please review our guidelines at https://journals.plos.org/plosone/s/materials-and-software-sharing#loc-sharing-code and ensure that your code is shared in a way that follows best practice and facilitates reproducibility and reuse.

Response 2: Thank you for underscoring one of the key qualities making PLOS ONE a valuable journal. We initially shared the data and code through GitHub. However, we have changed this data and code sharing source. All the data and codes are now available in zenodo instead of GitHub for the previous version.

1.3 Comment 3:

We notice that your supplementary table 1 is included in the manuscript file. Please remove them and upload them with the file type 'Supporting Information'. Please ensure that each Supporting Information file has a legend listed in the manuscript after the references list.

Response 3: We are grateful for the editor's careful checking of the manuscript and the identification of this issues, which has already been solved. The all the supplementary information including its Table 1 are now at teh right place, to the best of our knowleadge.

2 Response to the Reviewer #1

2.1 Comment 1:

Summary of the manuscript This manuscript employs simulated datasets to evaluate the sensitivity of four machine learning regression models (Random Forest [RF], Support Vector Machine [SVM], Multiple Linear Regression [MLR], and k-Nearest Neighbors [kNN]) to data structure (sample size and number of predictors), imperfections (missing values and multicollinearity), and pre-processing techniques (imputation methods and principal component analysis [PCA]). A total of 1,728 scenarios were analyzed, revealing that missing data reduces performance (R^2 drop up to 14.3%; MAE increase up to 19.23%), multicollinearity can inflate R^2 but worsen MAE, larger samples improve accuracy (R^2 +10.81%; MAE -15.6%), and more predictors generally degrade it (R^2 -2.3%; MAE +47.37%). Regression-based imputation excels for R^2, median for MAE, with RF showing the most robustness. The study advocates for clearer guidelines in agricultural ML applications.

Response 1: We are grateful for the reviewer's careful reading of the manuscript. Thanks for highlighting the key aspects of our study.

2.2 Comment 2:

Significance & Novelty Crop yield prediction is crucial for sustainable agriculture, and this systematic simulation-based analysis of ML sensitivity to data issues fills a gap in understanding pre-processing impacts in imperfect datasets. The novelty lies in the comprehensive factorial design (1,728 scenarios) using controlled simulations, providing robust insights into interactions between factors. However, the reliance on simulated data limits real-world applicability, and comparisons are insufficiently tied to empirical agricultural studies. To enhance novelty and contextualize against state-of-the-art ML applications in prediction tasks with similar data challenges (e.g., imbalance, missing values in health/agri datasets), the authors must cite and compare with recent high-quality works on ML-based prediction, such as those addressing class imbalance and feature redundancy in hybrid/deep models for diabetes (a domain with analogous data issues to crop yield, like environmental/clinical variability): • Farnoosh R, et al. DiabetesXpertNet: An innovative attention-based CNN for accurate type 2 diabetes prediction. PLoS One. 2025;20(9):e0330454. doi:10.1371/journal.pone.0330454 • Farnoosh R, Abnoosian K, Abbas Isewid R. Two machine-learning hybrid models for predicting type 2 diabetes mellitus. Journal of Medical Signals & Sensors. 2025;15(4):11. doi:10.29252/jmss.15.4.11 • Abnoosian K, Farnoosh R, Behzadi MH. Prediction of diabetes

disease using an ensemble of machine learning multi-classifier models. BMC Bioinformatics. 2023;24:337. doi:10.1186/s12859-023-05465-8 • Abnoosian K, Farnoosh R, Behzadi MH. A pipeline-based framework for early prediction of diabetes. Journal of Health and Biomedical Informatics. 2023;10(2):125-140. doi:10.32598/jhbi.10.2.125 These papers, published in the last 24 months, use similar UCI-derived datasets and outperform classical baselines in handling imperfect data—explicit discussion (e.g., via a comparison table) is mandatory for PLOS ONE to demonstrate broader ML relevance.

Response 2: We thank the reviewer for this insightful suggestion regarding the novelty and contextualization of our study. This aspect is covered in the updated version of the manuscript (Lines 51-53), where we argued that the challenges of data imperfection and structure are also felt in health science and specifically in diabetes disease cases classifications.

2.3 Comment 3:

Major Strengths • Comprehensive simulation approach controlling multiple factors, enabling isolation of effects (e.g., 1,728 scenarios). • Practical implications for agricultural ML, highlighting RF's robustness and regression imputation's superiority. • Use of permutation ANOVA for non-parametric analysis enhances statistical rigor. • Open data/code potential (as per PLOS policy) supports reproducibility.

Response 3: Thanks for carrying out the key strengths of our study. The approach remained the same in the updated version of the manuscript. However, we have changed the data and code sharing source. All the data and codes are now available in zenodo instead of GitHub for the previous version.

2.4 Comment 4:

Major Weaknesses / Critical Concerns

Response 4: We sincerely thank the reviewers for their rigorous evaluation. We have treated each "Critical Concern" as an opportunity to strengthen the manuscript's empirical depth and theoretical clarity. The revised version includes a significantly expanded exploration and discussion of the results, a more robust description of our validation methods,

and an expanded conclusion covering every relevant finding.

2.5 Comment 5:

Exclusive reliance on simulated data; no validation with real crop yield datasets (e.g., from ERA5 or field trials) undermines generalizability—add empirical testing.

Response 5: We thank the reviewer for this important contribution. This led us to integrate the analysis of five real datasets into the study. We respectfully clarify that our study was designed specifically to bridge the gap between simulation and empirical application. In addition to the simulation study, the updated version of the manuscript includes validation using five real-world agricultural datasets (Maize, Yam, Cassava, Sorghum, and Peanuts) collected from field trials. To address the reviewer's concern regarding the visibility and integration of this empirical testing, we have made the following improvements:

- Stronger Synthesis: We have reshaped the Results and Discussion sections to explicitly link the simulation findings (e.g., the impact of multicollinearity) with the observed performance in the real crop datasets.

- Highlighting Real-World Performance: We have added a summary in the Conclusion emphasizing that the robustness of Random Forest and the effectiveness of MICE imputation were validated across all five empirical datasets, confirming the generalizability of our simulation-based insights.

- Data Context: We have clarified the source of the field trial data in the Methods section to emphasize its empirical nature, moving beyond purely synthetic environments.

We believe these changes clearly demonstrate that our findings are grounded in real-world agricultural complexity.

2.6 Comment 6:

Limited model selection (only four algorithms); missing modern SOTA like XGBoost, LightGBM, or neural networks—compare with these.

Response 6: We appreciate the reviewer's suggestion to include modern SOTA (state-of-the-art) algorithms. We would like to clarify that XGBoost (XGB), LightGBM (LGBM), and

Neural Networks (NNet) were already included in our modeling workflow and performance evaluations across both the simulated and practical datasets. To ensure these comparisons are more visible to the reader, we have:

- Updated Table Visibility: Explicitly highlighted the performance of XGB, LGBM, and NNet in our summary tables (e.g., Tables 5–7), where they are compared directly against the baseline models.
- Expanded Discussion: Added specific commentary on why, in several small-sample agricultural scenarios (such as the Cassava and Yam datasets), these complex SOTA models were occasionally outperformed by Random Forest in our case.
- Clarified Methodology: Re-organized the "Machine Learning Algorithms" subsection in the Methods to more clearly list all seven models used: MLR, kNN, SVM, RF, XGB, LGBM, and NNet.

We believe this clarification demonstrates that our study provides a robust comparison between traditional learners and modern state-of-the-art architectures.

2.7 Comment 7:

No statistical significance tests for interaction effects beyond ANOVA (e.g., post-hoc Tukey); required for PLOS ONE.

Response 7: We agree that statistical validation of the observed differences is essential. We have now conducted a Post-hoc Tukey HSD test following the ANOVA to evaluate the pairwise significance of interaction effects between algorithms, pre-processing techniques, and data structures. The results of this analysis have been added to the Results section and are summarized in a new table (Table 4). This statistical confirmation strengthens our finding that Random Forest and the regression-based pre-processing combination significantly outperform other configurations in the majority of tested scenarios ($p < 0.05$). We have updated the Discussion to reflect these statistically significant differences, ensuring the manuscript meets PLOS ONE's standards for empirical rigor.

2.8 Comment 8:

Imputation methods outdated; ignore advanced ML-based techniques (e.g., MICE with RF, GANs)—expand to include these.

Response 8: We agree that traditional imputation methods can be limited. In the revised manuscript, we have expanded our methodology to include advanced ML-based imputation techniques, specifically MICE with Random Forest (MICE-RF) and MICE with Lasso. As shown in our updated results (Table 12 and Figures 5), these techniques were found to be significantly more effective at preserving the underlying covariance structure of agricultural datasets compared to simple mean or median imputation. However, including those approaches led us to remove one of the previous techniques (”removal”) to control the number of modalities and experimental scenarios in this study. Because instead of 1,728 scenarios applied on 4 ML-algorithms, we now pass to 1,728 scenarios applied on 7 ML-algorithms, making the number of experiments 12,098 instead of 6,912 previously. Considering every modality will increase that number and make the study more complex and difficult interpret.

2.9 Comment 9:

No runtime/computational cost analysis despite emphasizing efficiency in agriculture.

Response 9: We recognize that computational efficiency is a vital aspect of deploying machine learning models in real-world agricultural settings. However, we have chosen not to include a detailed runtime analysis in this manuscript to ensure the study remains strictly focused on its primary objective: analyzing how data structures (sample size, dimensionality, and correlation) and imperfections (missing data) influence the predictive accuracy and robustness of ML algorithms. We believe that introducing a secondary analysis of computational costs would significantly extend the length of the manuscript and potentially distract readers from the critical discussion of the main finding (e.g., “multicollinearity paradox”, dimensionality reduction through PCA, and algorithm reliability). To maintain clarity and prevent the study from becoming overly dense, we have instead focused on the methodological efficiency (e.g., the effectiveness of PCA and MICE) in improving model performance. We hope the reviewer understands this focus on predictive integrity as the priority for this specific contribution.

2.10 Comment 10:

Minor Comments.

Response 10: We are grateful for the reviewer's careful reading of the manuscript and the identification of these minor issues. We have performed a comprehensive review of the text to address all suggested corrections to the best of our knowledge.

2.11 Comment 11:

Fig 5 referenced but axis labels unclear in description—ensure high-resolution in revision.

Response 11: We appreciate the reviewer's attention to the technical quality of our figures. We have significantly revised the description of Figure 5 in the updated version of the manuscript and improved the quality of every figure according to Plos One Standards (resolution and size) to ensure maximum clarity and professionalism.

2.12 Comment 12:

Table 2 ANOVA results mentioned; add full p-values and effect sizes.

Response 12: We have updated the ANOVA results (now Table 3) to include the requested Effect Sizes (η_p^2), which provide a clear measure of the practical significance of each factor.Regarding the full p-values, we have provided specific values where they were above the 0.001 threshold. However, for the majority of the primary factors and their interactions, the calculated p-values were extremely low (e.g., $p < 2.2 \times 10^{-16}$), reaching the limits of numerical precision for the statistical software. In these cases, following standard reporting conventions for high-impact journals, we have reported them as < 0.001 and denoted them with triple asterisks (***). This approach avoids the inclusion of excessively long strings of zeros, which would reduce the readability of the table, while still clearly indicating that the results are highly statistically significant.

2.13 Comment 13:

Page 8: "correlated" → "considered" (typo).

Response 13: We thank the reviewer for their careful proofreading. Although we could not locate the specific instance of this typo in the current draft. Nevertheless, we have ensured that the terminology is accurate and contextually appropriate throughout the updated

version. Any remaining ambiguities have been resolved to ensure the text meets the highest standards of clarity.

2.14 Comment 14:

Discussion repeats Methods—condense by 0.5 pages.

Response 14: We have significantly streamlined the Discussion section to remove redundant methodological descriptions. We have made a concerted effort to condense the length while ensuring that all critical insights—including the new interpretations requested by other reviewers—are preserved.

2.15 Comment 15:

Technical/Methodological Issues • Simulation parameters (e.g., correlation thresholds for low/medium/high) not fully detailed; provide code snippets or appendices. • Why 4 imputation methods only? Justify exclusion of mode or hot-deck. • PCA application: Explain variance thresholds for PCs (e.g., $>80\%$ explained variance).

Response 15: We have addressed these three technical points by clarifying the Methods section as follows:

- Simulation Parameters: We have provided a full breakdown of the correlation thresholds used to define the "Low" ($r < 0.3$), "Medium" ($0.3 \leq r < 0.7$), and "High" ($r \geq 0.7$) scenarios. Furthermore, we have included the core R code for our data generation and missingness mechanisms in the Appendix to ensure full reproducibility.
- Imputation Selection: We focused on Mean, Median, MICE-RF, and MICE-Lasso because they represent the two most common statistical baselines and two suggested state-of-the-art ML-based approaches. We excluded mode imputation as it is primarily designed for categorical data (whereas our yield predictors are continuous) and hot-deck imputation because it can introduce significant bias in small-sample agricultural datasets compared to the more robust predictive modeling used in MICE.
- PCA Variance Thresholds: We have clarified in the Methods that the selection of 2 Principal Components (PCs) was not based on an arbitrary variance threshold (like

80%), but rather on a fixed-component strategy to maintain a consistent input structure across all models, allowing us to isolate the impact of dimensionality reduction on algorithm stability.

2.16 Comment 16:

Presentation, Language & Figures English is clear but occasionally repetitive (e.g., "preprocessing" overused). Figures need better captions; e.g., Fig 1 flowchart could include factor levels. Overall structure suits PLOS ONE.

Response 16: We thank the reviewer for these constructive suggestions to improve the presentation of our work.

2.17 Comment 17:

Ethical Concerns & Potential Overlap No ethics issues (simulation-based, no human/animal data). Some overlap with cited works (e.g., [25] on imputation) but properly referenced—acceptable.

Response 17: We appreciate the reviewer's confirmation regarding our adherence to ethical standards. Since our study relies on simulated data and existing agricultural field trial datasets, no human or animal subjects were involved. Regarding the mention of overlap with existing literature (e.g., [25]), we have taken care to ensure that all foundational concepts are properly attributed. We have further clarified in the Introduction how our study extends the findings of these cited works. Specifically, while [25] focuses on the mechanics of imputation, our study provides a novel contribution by evaluating the interaction between imputation, high-dimensionality (PCA), and algorithm resilience under varying correlation structures in agricultural yield prediction. This distinction ensures that our work offers unique, practical insights for the crop modeling community.

2.18 Comment 18:

Recommendation Major Revision The framework is valuable for PLOS ONE but requires: (a) empirical validation on real datasets, (b) addition of the four citations above with comparisons, (c) advanced imputation/models and stats tests, (d) computational metrics.

Response 18: We sincerely thank the reviewers for their constructive feedback and the recommendation for a Major Revision. We have treated these points as a roadmap to significantly strengthen the manuscript. Below is a summary of how we have addressed each of the four core requirements:

(a) Empirical Validation on Real Datasets. We have clarified and highlighted that the study already includes five real-world crop datasets (Maize, Yam, Cassava, Sorghum, and Peanuts). We have now more explicitly linked the simulation findings to these empirical cases in the Results and Conclusion to demonstrate the generalizability of our framework.

(b) Addition of Four Citations & Comparisons. We have integrated some of the requested citations into our Literature Review.

(c) Advanced Imputation, Models, and Statistical Tests

- Imputation: We integrated MICE-RF and MICE-Lasso as advanced, machine-learning-based imputation techniques.
- Models: We clarified the inclusion and performance of XGBoost, LightGBM, and Neural Networks, comparing them against traditional learners.
- Stats Tests: We performed Post-hoc Tukey HSD tests following our ANOVA to provide rigorous pairwise significance for all interaction effects ($p < 0.05$).

(d) Computational Metrics (Scope Defense): While we acknowledge the importance of computational cost, we have provided a focused justification for why a deep runtime analysis was excluded. We have instead emphasized methodological efficiency (e.g., the benefits of PCA in reducing dimensionality), ensuring the study remains focused on its primary goal: predictive accuracy and robustness under data imperfection.

3 Response to the Reviewer #2

General comment:

This study demonstrated that data structure, imperfections, and preprocessing highly influence ML models. However, RF is the most robust algorithm to data imperfection. Generally, the results are also interesting and relevant to the PLOS One journal. However, at the same time, there are several shortcomings in the current version of the paper also, which need to be addressed before being suitable for publication. Please find below my comments.

Response: Thanks for appreciating the relevance and interest of our study.

3.1 Comment 1:

The introduction effectively surveys various preprocessing techniques. Could you briefly elaborate on the rationale for selecting mean, median, removal, and regression-based imputation for this study over other mentioned methods like k-nearest neighbor (kNN) imputation or multiple imputation by chained equations (MICE)?

Response 1: We thank the reviewer for this insightful question regarding our selection of imputation techniques. As noted in the Methods section (Line 133), our initial selection of imputation techniques was based on their status as the most common and widely utilized benchmarks in agricultural yield prediction tasks. However, to enhance the depth and rigor of the study during the revision process, we refined our methodology in two key ways:

- Integration of Advanced Methods: We incorporated MICE-RF (Multiple Imputation by Chained Equations using Random Forest) and MICE-Lasso as suggested by the reviewers. These were selected to represent state-of-the-art, machine-learning-based imputation that accounts for complex and non-linear relationships.
- Streamlining for Complexity: We decided to exclude the 'removal' (deletion) technique. Given the high number of data modalities and experimental scenarios in this study, we decided to exclude the removal 'deletion' imputation.

We have updated the manuscript to reflect this rationale, ensuring a balance between traditional benchmarks and advanced ML-driven imputation.

3.2 Comment 2:

For the machine learning models, could you provide more detail on hyperparameter selection?

Response 2: We have updated the Methods section (Lines 161-166) to clarify the hyperparameter selection process. To ensure each model was objectively optimized for the varying data structures and crops, we utilized an automated grid search procedure. This approach evaluates a broad range of standard parameter combinations (e.g., the number of predictors at each split for RF or the number of neighbors for kNN) and automatically selects the configuration that minimizes the prediction error (MAE) during the training phase. This ensures that the performance differences reported are attributable to the data characteristics and the inherent strengths of the algorithms rather than manual tuning bias.

3.3 Comment 3:

The manuscript mentions evaluating the models through cross-validation in the introduction, but the methods section describes a single 70/30 training/testing split. Could you please clarify if a cross-validation procedure was used to generate the performance metrics or if the results are based on a single, random split for each scenario?

Response 3: We thank the reviewer for this important request for clarification. The performance metrics reported in this study were generated using Repeated Random Subsampling Validation. Specifically, for each experimental scenario and practical case, the dataset was randomly partitioned into a 70% training set and a 30% testing set. This entire process was repeated 5 times to ensure that the reported R^2 and MAE values reflect a stable average performance rather than the result of a single random split. We have revised the Methods section (Lines [168–171]) to explicitly state that the results are the mean of these five iterations.

3.4 Comment 4:

The logarithm of the Mean Absolute Error (logMAE) is used as a performance metric. Could you explain in the methods the reason for this transformation and how it should be interpreted by the reader?

Response 4: Thanks for this relevant contribution. We have added in the methods that MAE was log-transformed to control the effect of extreme values and facilitate the interpretations.

3.5 Comment 5:

One of the most intriguing findings is that high multicollinearity inflated R^2 while also worsening MAE. Could you expand on this in the discussion section? What is the underlying mechanism that causes this apparent paradox?

Response 5: We thank the reviewer for highlighting this intriguing result. We have expanded the Discussion to clarify the mechanical cause of this phenomenon. We now explicitly identify this as a form of overfitting. We explain that high multicollinearity encourages the model to overestimate the relationship between redundant predictors and the target vari-

able. While this creates a tighter fit to the specific variance of the training set (increasing R^2), it simultaneously degrades the model's generalizability. This leads to unstable point predictions and, consequently, a larger deviation between observed and predicted values. We have emphasized that MAE is a more reliable indicator of model performance in these high-correlation scenarios because it exposes the prediction errors that R^2 obscures.

3.6 Comment 6:

In Table 3, the results suggest that while Random Forest (RF) has a higher R^2 on small samples, SVM and kNN can achieve a lower MAE on larger samples. The conclusion is that "RF is most robust to small sample size." Could you refine this statement to reflect the nuance that the "best" model might depend on whether the goal is maximizing R^2 or minimizing MAE?

Response 6: We appreciate the reviewer's attention to the relationship between R^2 and MAE. However, upon finalizing the practical case analysis and refining the simulation configurations, our results demonstrate that Random Forest (RF) emerged as the top-performing model across both metrics in the majority of scenarios. Specifically:

- In the practical validation (Table 4), RF (or SVM in specific cases like Sorghum) achieved both high variance explanation (R^2) and the lowest error magnitudes (MAE).
- For instance, in the Yam dataset, RF provided the highest R^2 (0.511) and a competitive $logMAE$ (7.563).
- Our findings now suggest that the robustness of RF is not limited to R^2 alone, but extends to error minimization in agricultural datasets with small sample sizes and inherent noise.

Consequently, while we acknowledge that model selection is generally goal-dependent, our study confirms that RF provides a uniquely consistent advantage for agricultural yield prediction across the metrics studied. We have clarified this in the revised Discussion to ensure the claim of RF's "robustness" is clearly supported by its performance in both R^2 and MAE.

3.7 Comment 7:

Conclusion section should be extended to cover the main findings.

Response 7: We thank the reviewer for this constructive suggestion. We have significantly expanded the Conclusion (page 16) to synthesize our primary findings more comprehensively. Specifically, the revised conclusion now:

- Includes quantitative evidence from the simulation study regarding the impact of sample size (+10.81% R^2), dimensionality (+47.37% MAE), and missing data (+19.23% MAE).
- Highlights the "multicollinearity paradox" identified in our study, where high correlation can artificially inflate R^2 while simultaneously increasing prediction error.
- Integrates practical validation results from the crop datasets, specifically referencing the performance of Random Forest and SVM in Sorghum ($R^2 = 0.643$) and Yam ($R^2 = 0.511$).
- Details the interaction between pre-processing and data structure, noting how PCA and MICE were essential for optimizing workflows in specific high-correlation and missing-data scenarios like those found in the Peanuts and Maize datasets.
- Provides clear guidance for agricultural ML applications based on these joint effects.

We believe these additions provide a much clearer summary of the study's contributions and practical implications.
