# Supplementary material for: Sensitivity of machine learning regression models to data structure and quality in crop yield prediction
Source: PLoS One. 2026 Sep 1;21(9):e0353938. doi: 10.1371/journal.pone.0353938 (PMC13533432; doi:10.1371/journal.pone.0353938)
Supplement: Supplementary file 2 [file pone.0353938.s022.pdf]

Answers to reviewers' comments on Manuscript No. PONE-D-25-57279

June 9, 2026

Dear Professor Rajib Chowdhury,

On behalf of my co-authors, I would like to thank you for giving us an opportunity to submit a revised version of our manuscript entitled "**Sensitivity of machine learning regression models to data structure and quality in crop yield prediction**" to *PLOS ONE*. I would also like to thank you and the reviewers for the valuable time you devoted to proofreading the manuscript and for your valuable comments.

Your constructive comments have been very helpful in improving the quality and clarity of our manuscript. We provide point-by-point responses to the reviewers' comments below. The comments are reproduced in *italics*, and our responses are provided in regular (upright) font.

Sincerely,

On behalf of Zinzinhédo, Mitchozounnou, Salako, and Glèlè Kakaï.

1 Response to the Reviewer #2

1.1 Comment 1:

The study is well-structured with a large simulation component and a smaller practical validation component. Could you elaborate on how the practical validation results (where kNN and XGBoost were optimal in some cases) inform or challenge the main conclusion from the simulation study (which points to the general robustness of Random Forest)?

Response 1: We thank the reviewer for this insightful comment, which prompted us to sharpen the interpretation of our practical validation results by more explicitly linking each crop's optimal workflow to its specific data characteristics, an aspect that, while partially touched in the second paragraph of the Discussion, now receives the more rigorous treatment it deserves.
In fact, the practical validation results do not contradict but rather illustrate the simulation conclusion: RF's robustness refers to its **smallest performance degradation as data conditions deteriorate**, not to universal optimality under every specific configuration. In cases where kNN and XGBoost emerged as top performers (particularly Maize and Peanuts) it was not kNN or XGBoost that improved intrinsically, but rather the **pre-processing pipeline that reshaped the data geometry** in their favour: PCA-reduced orthogonal features favour distance-based learners, while high collinearity without dimensionality reduction smooths neighbourhood averaging in kNN's benefit. This is entirely consistent with our simulation finding that the **Algorithm $\times$ Imputation interaction carries the largest effect size** ($\eta_p^2 = 0.50$), confirming that algorithm performance cannot be evaluated independently of pre-processing decisions. RF therefore remains the safest and most robust default, while the practical results add the nuance that a well-matched pre-processing pipeline can unlock marginal advantages for alternative algorithms under specific data conditions.

1.2 Comment 2:

The introduction effectively surveys various preprocessing techniques. Could you briefly elaborate on the rationale for selecting mean, median, MICE-Lasso, and MICE-RF for this study over other mentioned methods like k-nearest neighbor (kNN) imputation or linear interpolation?

Response 2: We thank the reviewer for this relevant question, allowing us to make a clarification in the methodology. Mean and Median were selected for their status as the

most widely used univariate baseline methods in agricultural prediction tasks, providing a necessary reference point against which more sophisticated techniques can be meaningfully evaluated. MICE-Lasso and MICE-RF were selected because they explicitly account for the multivariate covariance structure of the data through a chained equations framework, a valuable property in agricultural datasets where missing values frequently occur across structurally related predictors, such as meteorological or remote sensing variables measured from the same source. Furthermore, given that our factorial design already encompasses 12,096 experimental scenarios across seven algorithms, four missing rates, three sample sizes, three predictor counts, and three collinearity levels, extending the number of imputation methods would have rendered the study computationally intractable and the results increasingly difficult to interpret in a coherent and scientifically meaningful way.

1.3 Comment 3:

You briefly draw a parallel to challenges in health science, specifically diabetes prediction. Could you expand on this comparison to better contextualize the novelty of your work? How do the data challenges in that domain reinforce the need for this specific study in agriculture?

Response 3: We thank the reviewer for this constructive suggestion, that helped us emphasized on the aspect in our introduction. The parallel drawn with health science was intended to highlight that the subjectivity of pre-processing choices is not unique to agriculture but reflects a broader methodological challenge across domains where observational data is inherently imperfect. Specifically, the cited works on diabetes prediction [45, 46] identified missing values, limited labeled data, high-dimensional feature spaces, and outliers as the primary obstacles to reliable predictive performance, challenges that are structurally analogous to those encountered in agricultural yield prediction, where weather variables, soil properties, and remote sensing indices are similarly subject to missingness, multicollinearity, and measurement noise across plots and seasons. These studies further demonstrated that classical baseline models were systematically outperformed when such imperfections were not adequately addressed, reinforcing the conclusion that pre-processing is not a peripheral step but a primary determinant of predictive integrity regardless of the application domain. What distinguishes the agricultural context (and constitutes the specific novelty of our contribution) is that, unlike clinical settings where data collection protocols are relatively standardised, field trial data are characterised by spatial heterogeneity, seasonal variability, and low observation-to-predictor ratios, making the consequences of suboptimal pre-processing choices considerably more severe and less predictable.

1.4 Comment 4:

For the machine learning models, could you provide more detail on the hyperparameter selection process? The manuscript mentions an "automated grid search procedure," which is appropriate, but detailing the range of parameters explored for each algorithm would significantly improve the reproducibility of your work.

Response 4: We entirely agree with the reviewer that complete transparency regarding hyperparameter configurations is vital for reproducibility. In accordance with your excellent suggestion, we have systematically documented the optimization parameters for all seven machine learning frameworks evaluated in this study. Rather than relegating this to a supplementary document, we have integrated these comprehensive configuration states directly into the core manuscript under Table 2. This table explicitly outlines the optimal hyperparameter values (including Random Forest mtry and num.trees; Support Vector Machine C and sigma; k-Nearest Neighbors k; XGBoost eta, *max_depth*, and nrounds; LightGBM parameters; and Neural Network size/decay) captured dynamically across each experimental pre-processing scenario. This provides an absolute, transparent log of the model states behind our performance metrics.

1.5 Comment 5:

The manuscript mentions evaluating models through cross-validation in the introduction, but the methods section describes a repeated 70/30 training/testing split iterated five times. Could you please clarify the terminology and procedure to ensure there is no ambiguity? Was this a repeated random sub-sampling validation?

Response 5: We thank the reviewer for this important request for clarification. We acknowledge the terminological inconsistency between the Introduction and the Methods section. The procedure employed was indeed a **repeated random subsampling validation**: each dataset was randomly partitioned into a 70% training set and a 30% testing set, and this procedure was iterated five times, with the final R^2 and MAE values reported as the mean across the five iterations. This approach differs from standard k-fold cross-validation, in which the data are partitioned into k non-overlapping folds with systematic rotation ensuring each observation appears in the test set exactly once. The reference to "cross-validation" in the Introduction was therefore imprecise, and we have corrected it to "repeated random subsampling validation" throughout the manuscript to ensure full terminological consistency and avoid any ambiguity for the reader.

1.6 Comment 6:

The logarithm of the Mean Absolute Error (logMAE) is used as a performance metric. Could you explain in the methods the reason for this transformation and how it should be interpreted by the reader, particularly in contrast to the more standard MAE?

Response 6: We thank the reviewer for this relevant question. Across 1,728 experimental scenarios spanning widely varying sample sizes, predictor counts, and missing rates, the raw MAE values exhibited a strongly right-skewed distribution with extreme values in poorly conditioned scenarios, which would have distorted visual comparisons of central tendencies across conditions; the logarithmic transformation therefore compresses this skewness, stabilises variance across groups, and renders graphical comparisons more interpretable without altering the ranking of methods or algorithms. Importantly, we added that logMAE preserves the ordinal structure of the original metric (a lower logMAE still unambiguously indicates better predictive accuracy) and all conclusions drawn from logMAE are directionally consistent with those that would be obtained from the untransformed MAE.

1.7 Comment 7:

The number of retained Principal Components (PCs) was set at 2, 3, and 5. What was the rationale for choosing these specific numbers? Were they based on a common heuristic, such as a certain threshold of explained variance, or were they chosen to represent different levels of dimensionality reduction?

Response 7: We thank the reviewer for this question. The number of retained Principal Components (2, 3, and 5) was chosen based on the cumulative proportion of explained variance, following a common heuristic in PCA-based pre-processing, as these levels generally captured a substantial amount of variability in the simulated datasets while achieving meaningful dimensionality reduction relative to the original predictor counts of 5, 8, and 12. Beyond the variance explanation criterion, these three levels were deliberately selected to represent a gradient of dimensionality reduction: from aggressive compression (2 PCs) to a near-complete retention of the original feature space (5 PCs), allowing us to systematically isolate the effect of the degree of dimensionality reduction on model performance across all experimental scenarios.

1.8 Comment 8:

One of your most intriguing findings is the "multicollinearity paradox," where high multicollinearity inflated R^2 while also worsening MAE. Could you expand on this in the discussion section? What is the underlying statistical mechanism that causes this apparent paradox, and how does it relate to model overfitting?

Response 8: We thank the reviewer for highlighting this finding, which we consider one of the most practically important contributions of this study. The multicollinearity paradox arises through two complementary mechanisms, both demonstrated step-by-step in the dedicated computational illustration included in the Discussion. First, highly correlated predictors create redundant pathways through which models minimise SS_E by fitting training noise as structural signal, producing inflated R^2 values that reflect overfitting rather than genuine predictive power, while MAE (computed as a simple average of absolute errors on unseen data) directly exposes the resulting prediction instability. Second, in agricultural systems where agroclimatic predictors such as temperature, solar radiation, soil moisture, and rainfall are structurally correlated through shared climatic drivers, their joint contribution amplifies the total variance of the response variable (SS_T), causing $R^2 = 1 - SS_E/SS_T$ to increase mechanically even when residuals also grow. This second mechanism is not a simulation artefact but a plausible real-world scenario reproduced by design in our simulation framework. Together, these two mechanisms confirm that R^2 and Error magnitude criteria (like MAE) must always be interpreted jointly in agricultural ML applications where multicollinearity is prevalent.

1.9 Comment 9:

In Table 4, the results suggest that while Random Forest (RF) often has a higher R^2 on small samples, other models like SVM and kNN can achieve a lower MAE on larger samples. Your conclusion is that "RF is the most robust algorithm." Could you refine this statement to reflect the nuance that the "best" model might depend on whether the primary goal is maximizing explained variance (R^2) or minimizing prediction error (MAE)?

Response 9: We thank the reviewer for this pertinent observation. We agree that the claim of RF's robustness deserves refinement to reflect the metric-dependent nuance visible in Table 5. Across all sample sizes, RF consistently achieved the highest R^2 values, including in the most challenging small-sample condition ($R^2 = 0.79$ at $n = 100$), where competing algorithms such as XGBoost (0.58) and NNet (0.54) degraded substantially, confirming RF's

superior robustness to data scarcity in terms of explained variance. However, as sample size increased to 500, SVM (logMAE = 2.31) and kNN (logMAE = 2.63) demonstrated increasingly competitive error minimization, narrowing the gap with RF. Despite this convergence, RF remained the best error minimizer across all sample sizes, achieving the lowest logMAE at $n = 100$ (logMAE = 2.37), $n = 300$ (logMAE = 2.37), and $n = 500$ (logMAE = 2.26), consistently outperforming all competing algorithms at every level of data availability. In cases where RF achived the best R^2 but the best MAE is provided with other algorithm (e.g., kNN, SVM, ...), we conclud that these two metrics are not sufficient to select the best algorithm.

1.10 Comment 10:

The practical validation in Table 9 shows that the optimal workflow for Peanuts (high collinearity, no missingness) was using 2 PCs with an XGBoost model. How does this finding support or modify your general conclusion that MICE-regression paired with 2 PCs is the most reliable workflow?

Response 10: We thank the reviewer for this insightful observation. The Peanuts result does not contradict but rather refines our general conclusion: since this dataset exhibited no missingness whatsoever, any imputation step was unnecessary, and the optimal workflow naturally reduced to its dimensionality reduction component alone (2 PCs) which is entirely consistent with our simulation finding that PCA provides the most effective noise filtering under high collinearity conditions. Our general conclusion therefore holds: when missingness is present, MICE-regression paired with 2 PCs is the most reliable workflow, while when data are complete but collinearity is high, 2 PCs alone with a boosting algorithm represents the natural and consistent specialisation of that same framework.

1.11 Comment 11:

The study provides an excellent analysis of model accuracy and error. Could you add a brief discussion on the computational costs associated with the different imputation methods and algorithms? A qualitative analysis of the trade-off between performance gains and computational expense would be very valuable for practitioners.

Response 11: We thank the reviewer for this highly practical suggestion. Evaluating computational costs is essential for translating ML models into accessible tools for agricul-

tural practitioners, who may operate under hardware or time constraints. In direct response to your comment, we have expanded the Discussion section to include a qualitative trade-off analysis balancing algorithmic performance gains against computational complexity.

1.12 Comment 12:

The conclusion effectively summarizes the key takeaways. Could you extend it to provide a more prescriptive set of guidelines for researchers? For example, based on your findings, what workflow would you recommend to a researcher who has a small dataset with moderate multicollinearity and a high percentage of missing values?

Response 12: We thank the reviewer for this constructive suggestion. We have extended the conclusion to include prescriptive guidelines tailored to specific data configurations: for small datasets with moderate multicollinearity and high missingness, we recommend MICE-RF imputation followed by 2 PCs and RF; for complete datasets with high collinearity, 2 PCs paired with XGBoost; and for low-missingness, low-collinearity data, preserving the original feature space with SVM or RF. These guidelines reinforce our central conclusion that pre-processing decisions must be strictly tailored to the dataset's specific correlation and missingness profile rather than treated as isolated choices.

2 Response to the Reviewer #3

General comment:

Summary: *The revised manuscript presents a comprehensive simulation-based analysis of the sensitivity of machine learning regression models to data structure, imperfections, and preprocessing strategies in the context of crop yield prediction. The authors have made substantial improvements in response to previous reviewer comments, including the integration of real-world datasets, inclusion of additional machine learning models (XGBoost, LightGBM, Neural Networks), incorporation of advanced imputation methods (MICE-based techniques), and the addition of post-hoc statistical testing. Overall, the manuscript is significantly strengthened and provides valuable insights into ML robustness under imperfect data conditions.*
Major Comments: *The authors have adequately addressed most of the major concerns raised in the previous review round. In particular:*

- *The inclusion of real-world datasets improves the practical relevance and generalizability of the study.*

- *The expansion of the model set to include modern state-of-the-art algorithms is appropriate and well-integrated.*

- *The addition of Tukey HSD post-hoc analysis strengthens the statistical rigor.*

- *The methodology is now more clearly described, including hyperparameter tuning and validation strategy.*

Response: We sincerely thank the reviewer for this thorough and encouraging assessment of the revised manuscript. We are pleased that the integration of real-world datasets, the expansion of the model set to include XGBoost, LightGBM, and Neural Networks, the incorporation of MICE-based imputation techniques, and the addition of Tukey HSD post-hoc analysis were received positively and recognised as meaningful contributions to the robustness and practical relevance of the study. We remain committed to addressing any remaining concerns raised in the specific comments that follow.

2.1 Comment 1:

Abstract inconsistency: *The Abstract contains an inconsistency regarding the number of machine learning models. While a specific number is mentioned, the listed models do not fully align with that count. Please revise the Abstract to ensure consistency between the stated number and the models included in the study.*

Response 1: We thank the reviewer for catching this inconsistency. The Abstract has been revised to explicitly include Neural Networks (NNet) in the list of evaluated algorithms, ensuring full consistency between the stated number of seven machine learning models (RF, SVM, MLR, XGBoost, LightGBM, NNet, and kNN) and those actually evaluated in the study.

2.2 Comment 2:

wording issue in Results: *In the Results section (around line 252), the authors state that Random Forest (RF) "achieved the lowest" performance, whereas the context and reported values in (table 5) indicate that RF actually achieved the highest performance. Please correct*

this wording to accurately reflect the results.

Response 2: We thank the reviewer for identifying this wording error. The sentence has been corrected to accurately reflect that RF maintained its superior performance across all predictor counts, while acknowledging that its metrics naturally declined as dimensionality increased (from $R^2 = 0.81$ at 5 predictors to $R^2 = 0.79$ at 12 predictors and from logMAE = 1.54 to logMAE = 3.11) but still outperformed all competing algorithms at every level of dimensionality.

2.3 Comment 3:

Clarity on validation strategy: *While the revised manuscript explains repeated random sub-sampling and 5 fold cross validation, the wording may still cause slight confusion. Consider clarifying the distinction between these procedures to improve readability.*

Response 3: We thank the reviewer for this observation. We acknowledge that the simultaneous mention of repeated random subsampling and 5-fold cross-validation in the manuscript may have introduced confusion, as these refer to two distinct validation strategies. To eliminate any ambiguity, we have revised the Methods section to clearly state that the procedure employed was exclusively a **repeated random subsampling validation**: each dataset was randomly partitioned into a 70% training set and a 30% testing set, and this process was iterated five times, with R^2 and MAE reported as the mean across the five iterations. This differs fundamentally from k-fold cross-validation, in which the data are partitioned into k non-overlapping folds with systematic rotation ensuring each observation appears in the test set exactly once. The reference to "5-fold cross-validation" has been removed throughout the manuscript and consistently replaced with "repeated random subsampling validation" to ensure terminological precision and readability.

Conclusion Comment:

Conclusion: *The manuscript is technically sound, methodologically improved, and relevant to the field. Only minor revisions related to clarity, consistency, and language are required before publication.*
Recommendation: *Minor Revision*

Response Conclusion: We sincerely thank the reviewer for this thorough and encouraging evaluation of the revised manuscript, and for the positive recommendation. We are committed to carefully addressing all remaining minor comments related to clarity, consistency, and language to ensure the manuscript meets the highest standards expected by PLOS ONE before final submission.

3 Response to the Reviewer #4

3.1 Comment 1:

The integration of an empirical dataset to evaluate performance introduces significant novelty to this work. To ensure the broader impact of the framework are immediately clear, this contribution should be mentioned in the Abstract. Doing so will clarify for the reader that the findings extend beyond simulated environments. Additionally, within the 'Validation through Practical Crop Datasets' subsection, the authors should address the potential for performance variability, stating how the pipeline's robustness might be affected when applied to different or more diverse datasets.

Response 1: We thank the reviewer for these two constructive suggestions, both of which have already been addressed in the revised manuscript. Regarding the first point, the Abstract has been updated to explicitly mention the empirical validation component, clarifying that the proposed framework was evaluated not only across 12,096 model-scenario evaluations on simulated datasets but also validated on five real-world agricultural datasets (Maize, Yam, Cassava, Sorghum, and Peanuts), thereby making the broader impact of the framework immediately clear to the reader. Regarding the second point, a dedicated sentence has been added at the end of the "Validation through Practical Crop Datasets" subsection acknowledging that performance variability should be anticipated when the framework is applied to more diverse datasets characterised by different agro-ecological conditions, crop physiologies, or data collection protocols, and that the guidelines proposed in this study should therefore be treated as evidence-based starting points rather than universal prescriptions. We believe these additions satisfactorily address both concerns raised by the reviewer.

3.2 Comment 2:

The methodology/resource regarding the empirical data characteristics requires further clarification. Currently, it is unclear how the specific attributes(Low missingness, No missingness) of the data were identified or achieved. Please provide a more transparent description of this process to support the technical rigor of the study and to ensure that the results are fully reproducible by other researchers.

Response 2: We thank the reviewer for this important observation. To ensure full transparency and reproducibility, we have explicitly defined the missingness classification thresholds used to characterise the practical datasets. Specifically, datasets with 0% missing observations are classified as "No missingness"; those with missing rates in $[0\%, 7\%[$ as "Low missingness"; those in $[7\%, 15\%[$ as "Moderate missingness"; and those exceeding 15% as "High missingness". Applying these thresholds consistently: Peanuts (0%) is classified as No missingness, Cassava (3.85%) and Sorghum (5.77%) as Low missingness, and Yam (8.65%) and Maize (8.65%) as Moderate missingness, fully consistent with the classifications reported in Table 9 and the analyses conducted throughout the study. These thresholds have been explicitly added to the Methods section to ensure full reproducibility.

3.3 Comment 3:

While the content is logically sound, the use of 'Practice' as a heading/subheading is too vague and fails to convey the conceptual focus of the subsection (I recommend replacing it with a more descriptive title such as 'Practical Implementation').

Response 3: We thank the reviewer for this stylistic suggestion. The heading "Practice" has been replaced throughout the manuscript with "Practical Implementation", which more accurately conveys the conceptual focus of the subsection and better aligns with the scientific tone expected by PLOS ONE.

3.4 Comment 4:

Furthermore, the paragraphs in Results section appear fragmented due to a lack of consistent heading and subheading conventions. Please adopt a standardized hierarchy, similar to the formatting used in the 'Methodology' section to ensure structural coherence throughout the

manuscript.

Response 4: We thank the reviewer for this constructive observation. In the revised manuscript, we have adopted a standardised heading hierarchy throughout the Results section, consistent with the formatting conventions used in the Methodology section. Specifically, the Results section is now organised as follows: the first subsection presents the overall "Performance of ML Algorithms"; the second subsection, entitled "Analysis of Sensitivity", groups all sensitivity analyses under a consistent sub-subsection hierarchy covering "Sensitivity of ML to Data Structure", "Sensitivity of ML to Data Imperfection", "Sensitivity of ML to Pre-processing Methods", and "Other Interactions between Data Structure, Imperfection, and Pre-processing"; the third subsection addresses "Optimal Actions on the Common Challenges Faced in Imperfect Datasets Pre-processing for ML-based Prediction"; and the fourth subsection presents the "Validation through Practical Crop Datasets". This restructuring eliminates the fragmentation previously observed and ensures full structural coherence throughout the manuscript.

4 Response to the Reviewer #5

General comment:

The authors have addressed many of the major concerns raised in the first review round, especially by expanding the model set, adding empirical crop datasets, strengthening the statistical analysis, and improving data/code availability. However, several reviewer/editor concerns are only partially addressed, and a few new inconsistencies introduced during revision should be corrected before the manuscript is accepted.

Response: We sincerely thank the reviewer for this balanced and thorough assessment of the revised manuscript, and for recognising the improvements made in response to the first round of review, particularly the expansion of the model set, the integration of empirical crop datasets, the strengthening of the statistical analysis, and the improvement of data and code availability. We acknowledge that several concerns remain partially addressed and that a few inconsistencies were inadvertently introduced during revision; we treat each of the specific comments that follow as an opportunity to bring the manuscript to the level of rigour and consistency required for publication in PLOS ONE, and we provide point-by-point responses accordingly.

4.1 Comment 1:

The Abstract states that the study evaluated "RF, SVM, MLR, XGBoost, LightBoost, and kNN," which lists only six algorithms and omits Neural Networks, although the manuscript later states that seven algorithms were used. It also uses "LightBoost," whereas the Methods use Light Gradient Boosting Machine / LGBM. This should be corrected throughout.

Response 1: We thank the reviewer for carefully identifying these inconsistencies. The Abstract has been corrected to list all seven algorithms evaluated in the study, RF, SVM, MLR, XGBoost, LightGBM, NNet, and kNN, ensuring full consistency with the Methods section and the stated number of seven algorithms. Additionally, the erroneous term "LightBoost" has been replaced with "LightGBM" (Light Gradient Boosting Machine) throughout the entire manuscript, including the Abstract, Results, and Discussion sections, to ensure terminological consistency and accuracy.

4.2 Comment 2:

The manuscript states that 1,728 combinations were run for each ML algorithm and that the seven algorithms produced 12,096 scenarios in total. However, the Abstract says "1,728 scenarios across the seven ML algorithms," which underreports the total experimental runs. The authors should clearly distinguish between: "1,728 data/pre-processing scenarios per algorithm" and "12,096 model-scenario evaluations in total."

Response 2: We thank the reviewer for identifying this important underreporting in the Abstract. The sentence has been corrected to clearly distinguish between the number of data and pre-processing scenarios per algorithm and the total number of model-scenario evaluations conducted across all seven algorithms.

4.3 Comment 3:

Hyperparameter tuning needs more detail. The authors should add a supplementary table listing the tuned hyperparameters and ranges for each model, for example: RF mtry, number of trees; SVM C, sigma; kNN k; XGBoost eta, max_depth, nrounds; LGBM parameters; and NNet hidden units/decay. This is important for reproducibility.

Response 3: We thank the reviewer for this constructive recommendation. We completely agree that providing the exact search spaces is essential for absolute experimental transparency and reproducibility. In the revised manuscript, we have expanded the methodology section to explicitly document (Table 2) the parameter ranges and fixed tuning constants established for each algorithm during our optimization phase. Specifically, Table 2 details the following grid configurations evaluated via repeated random subsampling validation:

- **Random Forest (RF):** The number of trees was fixed at 470; the splitting feature candidate parameter (*mtry*) was searched across $\{2, \lfloor\sqrt{p}\rfloor, p\}$, where p is the number of active features or principal components; and the minimum node size was evaluated at $\{1, 5\}$ under the variance split rule.

- **Support Vector Machines (SVM Radial):** The Radial Basis Function (RBF) kernel parameter σ was fixed at 0.1, while the cost regularization parameter C was optimized across the grid $\{1, 4\}$.

- **k-Nearest Neighbors (kNN):** The neighborhood parameter k was optimized using an odd-integer sequence ranging from 1 up to $\min(20, n_{\text{train}} - 1)$.

- **XGBoost:** The maximum tree depth (*max_depth*) was explored across $\{3, 6\}$; the learning rate (*eta*) across $\{0.05, 0.1\}$; and the row subsampling fraction (*subsample*) across $\{0.8, 1.0\}$, evaluated over up to 100 iterations with an early stopping round threshold of 10.

- **LightGBM:** Configured under a learning rate of 0.1 and run for 50 iterations focused on minimizing root mean squared error (RMSE).

- **Neural Networks (NNet):** The size of the hidden layer (*size*) was evaluated across $\{3, 5\}$ in combination with a weight decay parameter set at 0.1.

4.4 Comment 4:

The manuscript would benefit from a broader regression-evaluation framework. Although R^2 and MAE are useful, the authors should consider adding MAPE, as it provides an intuitive percentage-based measure of prediction error and facilitates comparison across crops and yield scales. RMSE or normalized RMSE could also be reported to strengthen the performance comparison.

Response 4: We thank the reviewer for this constructive suggestion. In the revised manuscript, we have extended the evaluation framework beyond R^2 and MAE by incorporating two additional performance metrics: the Mean Absolute Percentage Error (MAPE) and the Root Mean Squared Error (RMSE), the latter reported on the logarithmic scale (logRMSE) to control the effect of extreme values across the wide range of simulated yield magnitudes, consistently with the log-transformation already applied to MAE. We acknowledge that including the full set of metrics directly in the main text would substantially increase the length of the manuscript and risk distracting readers from the primary objective of the study, which is to systematically assess the sensitivity of ML algorithms to data structure and imperfection rather than to provide an exhaustive metric-by-metric comparison. For this reason, these additional metrics are reported across all experimental conditions (data structure, imperfection, pre-processing methods, and their interactions) in four dedicated supplementary tables (S2 Table to S5 Table), which mirror the structure of the main results tables and allow direct comparison across algorithms, imputation methods, and dimensionality reduction strategies. MAPE in particular facilitates intuitive interpretation of prediction error as a percentage of the observed yield value, and supports comparison across the five empirical crop datasets which operate on different yield scales. The conclusions drawn from logRMSE and MAPE are directionally consistent with those obtained from R^2 and logMAE, confirming the robustness of our findings across multiple evaluation frameworks.

4.5 Comment 5:

Since "sensitivity" is central to the manuscript title, the sensitivity analysis should be strengthened. In addition to missingness, multicollinearity, sample size, and predictor number, the authors should consider testing model robustness under controlled perturbations such as Gaussian noise injection into the input predictors. The manuscript should also acknowledge uncertainty-aware approaches, such as quantile regression evaluated with pinball loss, either as an additional analysis or as a clear direction for future work.

Response 5: We thank the reviewer for these stimulating methodological suggestions, which we have carefully considered. Regarding Gaussian noise injection, we fully agree that testing model robustness under controlled perturbations of input predictors would constitute a meaningful extension of the sensitivity framework proposed in this study. However, our factorial design already encompasses 12,096 model-scenario evaluations across seven algorithms, four missing rates, three sample sizes, three predictor counts, three collinearity levels, four imputation methods, and four dimensionality reduction configurations. Incorporating additional noise perturbation levels would substantially increase this experimental

complexity, rendering the study computationally intractable and the results increasingly difficult to interpret in a coherent and scientifically meaningful way. We therefore position Gaussian noise injection as a clearly prioritised direction for future work, explicitly acknowledged in the revised "Limitations and Future Work" section. Regarding uncertainty-aware approaches, we equally agree that probabilistic performance assessment (such as quantile regression evaluated with pinball loss) represents an important and underexplored dimension in agricultural ML applications, particularly under imperfect data conditions where point predictions may obscure meaningful uncertainty in yield forecasts. Since our study focuses exclusively on regression point prediction under controlled data imperfection, incorporating a full uncertainty quantification framework would constitute a distinct and substantial research contribution beyond the scope of the present work. We have therefore added both Gaussian noise injection and uncertainty-aware evaluation (including quantile regression and pinball loss) as explicit and prioritised future directions in the revised "Limitations and Future Work" section, acknowledging that these extensions would significantly broaden the sensitivity framework proposed here and open promising avenues for subsequent investigations.

4.6 Comment 6:

A dedicated section titled "Limitations and Future Work" should be added before the Conclusion. This section should clearly discuss the main limitations of the study, including the reliance on simulated data, the limited number of real-world crop datasets, the selected missingness mechanism, the restricted set of algorithms, and the limited evaluation metrics.

Response 6: We thank the reviewer for this constructive structural suggestion, which we have fully implemented. A dedicated "Limitations and Future Work" section has been added to the revised manuscript immediately before the Conclusion, explicitly addressing the following points. First, although carefully designed, the simulation framework relies on synthetic data that may not fully capture the complexity of real field environments, and the empirical validation is restricted to five crop datasets from a specific agro-ecological context in Benin, limiting immediate generalisability to other crops and regions. Furthermore, while seven algorithms spanning a broad spectrum of learning paradigms were evaluated, deep learning architectures such as Long Short-Term Memory (LSTM) networks and Convolutional Neural Networks (CNN) were not considered and may offer additional robustness under specific data conditions. Regarding the evaluation framework, although R^2, MAE, logRMSE, and MAPE were reported, future work should explore uncertainty-aware metrics such as quantile regression assessed with pinball loss, which would provide a more compre-

hensive picture of predictive reliability under imperfect data conditions. Finally, the sensitivity analysis could be further extended by testing model robustness under controlled input perturbations such as Gaussian noise injection, and hyperparameter optimisation strategies beyond grid search (such as Bayesian optimisation) may alter algorithm performance rankings under specific conditions and deserve systematic investigation in future studies.
