# Supplementary material for: Sensitivity of machine learning regression models to data structure and quality in crop yield prediction
Source: PLoS One. 2026 Sep 1;21(9):e0353938. doi: 10.1371/journal.pone.0353938 (PMC13533432; doi:10.1371/journal.pone.0353938)
Supplement: Supplementary file 3 [file pone.0353938.s019.docx]

PLOS ONE – Peer Review Report Manuscript Number: PONE-D-25-57279 Manuscript Title: Sensitivity of machine learning regression models to data structure and quality in crop yield prediction. Short Title: Sensitivity of machine learning regression models to data structure and quality. Date: November 15, 2025 Reviewer: Anonymous (invited reviewer)

1. Summary of the manuscript This manuscript employs simulated datasets to evaluate the sensitivity of four machine learning regression models (Random Forest [RF], Support Vector Machine [SVM], Multiple Linear Regression [MLR], and k-Nearest Neighbors [kNN]) to data structure (sample size and number of predictors), imperfections (missing values and multicollinearity), and pre-processing techniques (imputation methods and principal component analysis [PCA]). A total of 1,728 scenarios were analyzed, revealing that missing data reduces performance (R² drop up to 14.3%; MAE increase up to 19.23%), multicollinearity can inflate R² but worsen MAE, larger samples improve accuracy (R² +10.81%; MAE -15.6%), and more predictors generally degrade it (R² -2.3%; MAE +47.37%). Regression-based imputation excels for R², median for MAE, with RF showing the most robustness. The study advocates for clearer guidelines in agricultural ML applications.
2. Significance & Novelty Crop yield prediction is crucial for sustainable agriculture, and this systematic simulation-based analysis of ML sensitivity to data issues fills a gap in understanding pre-processing impacts in imperfect datasets. The novelty lies in the comprehensive factorial design (1,728 scenarios) using controlled simulations, providing robust insights into interactions between factors. However, the reliance on simulated data limits real-world applicability, and comparisons are insufficiently tied to empirical agricultural studies. To enhance novelty and contextualize against state-of-the-art ML applications in prediction tasks with similar data challenges (e.g., imbalance, missing values in health/agri datasets), the authors must cite and compare with recent high-quality works on ML-based prediction, such as those addressing class imbalance and feature redundancy in hybrid/deep models for diabetes (a domain with analogous data issues to crop yield, like environmental/clinical variability): • Farnoosh R, et al. DiabetesXpertNet: An innovative attention-based CNN for accurate type 2 diabetes prediction. PLoS One. 2025;20(9):e0330454. doi:10.1371/journal.pone.0330454 • Farnoosh R, Abnoosian K, Abbas Isewid R. Two machine-learning hybrid models for predicting type 2 diabetes mellitus. Journal of Medical Signals & Sensors. 2025;15(4):11. doi:10.29252/jmss.15.4.11 • Abnoosian K, Farnoosh R, Behzadi MH. Prediction of diabetes disease using an ensemble of machine learning multi-classifier models. BMC Bioinformatics. 2023;24:337. doi:10.1186/s12859-023-05465-8 • Abnoosian K, Farnoosh R, Behzadi MH. A pipeline-based framework for early prediction of diabetes. Journal of Health and Biomedical Informatics. 2023;10(2):125-140. doi:10.32598/jhbi.10.2.125 These papers, published in the last 24 months, use similar UCI-derived datasets and outperform classical baselines in handling imperfect data—explicit discussion (e.g., via a comparison table) is mandatory for PLOS ONE to demonstrate broader ML relevance.
3. Major Strengths • Comprehensive simulation approach controlling multiple factors, enabling isolation of effects (e.g., 1,728 scenarios). • Practical implications for agricultural ML, highlighting RF's robustness and regression imputation's superiority. • Use of permutation ANOVA for non-parametric analysis enhances statistical rigor. • Open data/code potential (as per PLOS policy) supports reproducibility.
4. Major Weaknesses / Critical Concerns
5. Exclusive reliance on simulated data; no validation with real crop yield datasets (e.g., from ERA5 or field trials) undermines generalizability—add empirical testing.
6. Limited model selection (only four algorithms); missing modern SOTA like XGBoost, LightGBM, or neural networks—compare with these.
7. No statistical significance tests for interaction effects beyond ANOVA (e.g., post-hoc Tukey); required for PLOS ONE.
8. Imputation methods outdated; ignore advanced ML-based techniques (e.g., MICE with RF, GANs)—expand to include these.
9. No runtime/computational cost analysis despite emphasizing efficiency in agriculture.
10. Minor Comments
11. Fig 5 referenced but axis labels unclear in description—ensure high-resolution in revision.
12. Table 2 ANOVA results mentioned; add full p-values and effect sizes.
13. Page 8: "correlated" → "considered" (typo).
14. Discussion repeats Methods—condense by 0.5 pages.
15. Technical/Methodological Issues • Simulation parameters (e.g., correlation thresholds for low/medium/high) not fully detailed; provide code snippets or appendices. • Why 4 imputation methods only? Justify exclusion of mode or hot-deck. • PCA application: Explain variance thresholds for PCs (e.g., >80% explained variance).
16. Presentation, Language & Figures English is clear but occasionally repetitive (e.g., "pre-processing" overused). Figures need better captions; e.g., Fig 1 flowchart could include factor levels. Overall structure suits PLOS ONE.
17. Ethical Concerns & Potential Overlap No ethics issues (simulation-based, no human/animal data). Some overlap with cited works (e.g., [25] on imputation) but properly referenced—acceptable.
18. Recommendation Major Revision The framework is valuable for PLOS ONE but requires: (a) empirical validation on real datasets, (b) addition of the four citations above with comparisons, (c) advanced imputation/models and stats tests, (d) computational metrics.

Confidential Comments to the Editor Solid methodological contribution with high citation potential in agri-ML, but simulation-only limits impact—insist on real data addition. Title/claims fine, but integrating medical ML parallels (via suggested cites) broadens appeal. Current: mid-tier PLOS ONE; post-revision: strong accept.
