# Supplementary material for: Sensitivity of machine learning regression models to data structure and quality in crop yield prediction
Source: PLoS One. 2026 Sep 1;21(9):e0353938. doi: 10.1371/journal.pone.0353938 (PMC13533432; doi:10.1371/journal.pone.0353938)
Supplement: Peer review history

# Peer review: Decision Letter 0

**DOI:** 10.1371/journal.pone.0353938.r001

**Authors:** Rajib Chowdhury

19 Nov 2025

Dear Dr. Zinzinhédo,

Thank you for submitting your manuscript to PLOS ONE. After careful consideration, we feel that it has merit but does not fully meet PLOS ONE’s publication criteria as it currently stands. Therefore, we invite you to submit a revised version of the manuscript that addresses the points raised during the review process.

Please submit your revised manuscript by Jan 03 2026 11:59PM. If you will need more time than this to complete your revisions, please reply to this message or contact the journal office at plosone@plos.org. When you're ready to submit your revision, log on to https://www.editorialmanager.com/pone/ and select the 'Submissions Needing Revision' folder to locate your manuscript file.

- A rebuttal letter that responds to each point raised by the academic editor and reviewer(s). You should upload this letter as a separate file labeled 'Response to Reviewers'.
- A marked-up copy of your manuscript that highlights changes made to the original version. You should upload this as a separate file labeled 'Revised Manuscript with Track Changes'.
- An unmarked version of your revised paper without tracked changes. You should upload this as a separate file labeled 'Manuscript'.

If you would like to make changes to your financial disclosure, please include your updated statement in your cover letter. Guidelines for resubmitting your figure files are available below the reviewer comments at the end of this letter.

If applicable, we recommend that you deposit your laboratory protocols in protocols.io to enhance the reproducibility of your results. Protocols.io assigns your protocol its own identifier (DOI) so that it can be cited independently in the future. For instructions see: https://journals.plos.org/plosone/s/submission-guidelines#loc-laboratory-protocols. Additionally, PLOS ONE offers an option for publishing peer-reviewed Lab Protocol articles, which describe protocols hosted on protocols.io. Read more information on sharing protocols at https://plos.org/protocols?utm_medium=editorial-email&utm_source=authorletters&utm_campaign=protocols.

We look forward to receiving your revised manuscript.

Kind regards,

Rajib Chowdhury, M.Sc.; MPH

Academic Editor

PLOS ONE

Journal requirements:

When submitting your revision, we need you to address these additional requirements.

1. Please ensure that your manuscript meets PLOS ONE's style requirements, including those for file naming. The PLOS ONE style templates can be found at

https://journals.plos.org/plosone/s/file?id=wjVg/PLOSOne_formatting_sample_main_body.pdf and

https://journals.plos.org/plosone/s/file?id=ba62/PLOSOne_formatting_sample_title_authors_affiliations.pdf

2. Please note that PLOS One has specific guidelines on code sharing for submissions in which author-generated code underpins the findings in the manuscript. In these cases, we expect all author-generated code to be made available without restrictions upon publication of the work. Please review our guidelines at https://journals.plos.org/plosone/s/materials-and-software-sharing#loc-sharing-code and ensure that your code is shared in a way that follows best practice and facilitates reproducibility and reuse.

3. We notice that your supplementary table 1 is included in the manuscript file. Please remove them and upload them with the file type 'Supporting Information'. Please ensure that each Supporting Information file has a legend listed in the manuscript after the references list.

If the reviewer comments include a recommendation to cite specific previously published works, please review and evaluate these publications to determine whether they are relevant and should be cited. There is no requirement to cite these works unless the editor has indicated otherwise.

[Note: HTML markup is below. Please do not edit.]

Reviewers' comments:

Reviewer's Responses to Questions

**Comments to the Author**

1. Is the manuscript technically sound, and do the data support the conclusions?

Reviewer #1: Yes

Reviewer #2: Yes

**********

2. Has the statistical analysis been performed appropriately and rigorously?

Reviewer #1: Yes

Reviewer #2: Yes

**********

3. Have the authors made all data underlying the findings in their manuscript fully available?

The PLOS Data policy

Reviewer #1: Yes

Reviewer #2: Yes

**********

4. Is the manuscript presented in an intelligible fashion and written in standard English?

Reviewer #1: Yes

Reviewer #2: Yes

**********

Reviewer #1: PLOS ONE – Peer Review Report Manuscript Number: PONE-D-25-57279 Manuscript Title: Sensitivity of machine learning regression models to data structure and quality in crop yield prediction. Short Title: Sensitivity of machine learning regression models to data structure and quality. Date: November 15, 2025 Reviewer: Anonymous (invited reviewer)

1. Summary of the manuscript This manuscript employs simulated datasets to evaluate the sensitivity of four machine learning regression models (Random Forest [RF], Support Vector Machine [SVM], Multiple Linear Regression [MLR], and k-Nearest Neighbors [kNN]) to data structure (sample size and number of predictors), imperfections (missing values and multicollinearity), and pre-processing techniques (imputation methods and principal component analysis [PCA]). A total of 1,728 scenarios were analyzed, revealing that missing data reduces performance (R² drop up to 14.3%; MAE increase up to 19.23%), multicollinearity can inflate R² but worsen MAE, larger samples improve accuracy (R² +10.81%; MAE -15.6%), and more predictors generally degrade it (R² -2.3%; MAE +47.37%). Regression-based imputation excels for R², median for MAE, with RF showing the most robustness. The study advocates for clearer guidelines in agricultural ML applications.

2. Significance & Novelty Crop yield prediction is crucial for sustainable agriculture, and this systematic simulation-based analysis of ML sensitivity to data issues fills a gap in understanding pre-processing impacts in imperfect datasets. The novelty lies in the comprehensive factorial design (1,728 scenarios) using controlled simulations, providing robust insights into interactions between factors. However, the reliance on simulated data limits real-world applicability, and comparisons are insufficiently tied to empirical agricultural studies. To enhance novelty and contextualize against state-of-the-art ML applications in prediction tasks with similar data challenges (e.g., imbalance, missing values in health/agri datasets), the authors must cite and compare with recent high-quality works on ML-based prediction, such as those addressing class imbalance and feature redundancy in hybrid/deep models for diabetes (a domain with analogous data issues to crop yield, like environmental/clinical variability): • Farnoosh R, et al. DiabetesXpertNet: An innovative attention-based CNN for accurate type 2 diabetes prediction. PLoS One. 2025;20(9):e0330454. doi:10.1371/journal.pone.0330454 • Farnoosh R, Abnoosian K, Abbas Isewid R. Two machine-learning hybrid models for predicting type 2 diabetes mellitus. Journal of Medical Signals & Sensors. 2025;15(4):11. doi:10.29252/jmss.15.4.11 • Abnoosian K, Farnoosh R, Behzadi MH. Prediction of diabetes disease using an ensemble of machine learning multi-classifier models. BMC Bioinformatics. 2023;24:337. doi:10.1186/s12859-023-05465-8 • Abnoosian K, Farnoosh R, Behzadi MH. A pipeline-based framework for early prediction of diabetes. Journal of Health and Biomedical Informatics. 2023;10(2):125-140. doi:10.32598/jhbi.10.2.125 These papers, published in the last 24 months, use similar UCI-derived datasets and outperform classical baselines in handling imperfect data—explicit discussion (e.g., via a comparison table) is mandatory for PLOS ONE to demonstrate broader ML relevance.

3. Major Strengths • Comprehensive simulation approach controlling multiple factors, enabling isolation of effects (e.g., 1,728 scenarios). • Practical implications for agricultural ML, highlighting RF's robustness and regression imputation's superiority. • Use of permutation ANOVA for non-parametric analysis enhances statistical rigor. • Open data/code potential (as per PLOS policy) supports reproducibility.

4. Major Weaknesses / Critical Concerns

5. Exclusive reliance on simulated data; no validation with real crop yield datasets (e.g., from ERA5 or field trials) undermines generalizability—add empirical testing.

6. Limited model selection (only four algorithms); missing modern SOTA like XGBoost, LightGBM, or neural networks—compare with these.

7. No statistical significance tests for interaction effects beyond ANOVA (e.g., post-hoc Tukey); required for PLOS ONE.

8. Imputation methods outdated; ignore advanced ML-based techniques (e.g., MICE with RF, GANs)—expand to include these.

9. No runtime/computational cost analysis despite emphasizing efficiency in agriculture.

10. Minor Comments

11. Fig 5 referenced but axis labels unclear in description—ensure high-resolution in revision.

12. Table 2 ANOVA results mentioned; add full p-values and effect sizes.

13. Page 8: "correlated" → "considered" (typo).

14. Discussion repeats Methods—condense by 0.5 pages.

15. Technical/Methodological Issues • Simulation parameters (e.g., correlation thresholds for low/medium/high) not fully detailed; provide code snippets or appendices. • Why 4 imputation methods only? Justify exclusion of mode or hot-deck. • PCA application: Explain variance thresholds for PCs (e.g., >80% explained variance).

16. Presentation, Language & Figures English is clear but occasionally repetitive (e.g., "pre-processing" overused). Figures need better captions; e.g., Fig 1 flowchart could include factor levels. Overall structure suits PLOS ONE.

17. Ethical Concerns & Potential Overlap No ethics issues (simulation-based, no human/animal data). Some overlap with cited works (e.g., [25] on imputation) but properly referenced—acceptable.

18. Recommendation Major Revision The framework is valuable for PLOS ONE but requires: (a) empirical validation on real datasets, (b) addition of the four citations above with comparisons, (c) advanced imputation/models and stats tests, (d) computational metrics.

Reviewer #2: This study demonstrated that data structure, imperfections, and preprocessing highly influence ML models. However, RF is the most robust algorithm to data imperfection. Generally, the results are also interesting and relevant to the PLOS One journal. However, at the same time, there are several shortcomings in the current version of the paper also, which need to be addressed before being suitable for publication. Please find below my comments.

The introduction effectively surveys various preprocessing techniques. Could you briefly elaborate on the rationale for selecting mean, median, removal, and regression-based imputation for this study over other mentioned methods like k-nearest neighbor (kNN) imputation or multiple imputation by chained equations (MICE)?

For the machine learning models, could you provide more detail on hyperparameter selection?

The manuscript mentions evaluating the models through cross-validation in the introduction, but the methods section describes a single 70/30 training/testing split. Could you please clarify if a cross-validation procedure was used to generate the performance metrics or if the results are based on a single, random split for each scenario?

The logarithm of the Mean Absolute Error (logMAE) is used as a performance metric. Could you explain in the methods the reason for this transformation and how it should be interpreted by the reader?

One of the most intriguing findings is that high multicollinearity inflated R² while also worsening MAE. Could you expand on this in the discussion section? What is the underlying mechanism that causes this apparent paradox?

In Table 3, the results suggest that while Random Forest (RF) has a higher R² on small samples, SVM and kNN can achieve a lower MAE on larger samples. The conclusion is that "RF is most robust to small sample size." Could you refine this statement to reflect the nuance that the "best" model might depend on whether the goal is maximizing R² or minimizing MAE?

Conclusion section should be extended to cover the main findings

**********

what does this mean?). If published, this will include your full peer review and any attached files.

If you choose “no”, your identity will remain anonymous but your review may still be made public.

**Do you want your identity to be public for this peer review?** For information about this choice, including consent withdrawal, please see our Privacy Policy

Reviewer #1: **Yes:** ok

Reviewer #2: **Yes:** Ali Salem

**********

[NOTE: If reviewer comments were submitted as an attachment file, they will be attached to this email and accessible via the submission site. Please log into your account, locate the manuscript record, and check for the action link "View Attachments". If this link does not appear, there are no attachment files.]

To ensure your figures meet our technical requirements, please review our figure guidelines: https://journals.plos.org/plosone/s/figures

You may also use PLOS’s free figure tool, NAAS, to help you prepare publication quality figures: https://journals.plos.org/plosone/s/figures#loc-tools-for-figure-preparation.

NAAS will assess whether your figures meet our technical requirements by comparing each figure against our figure specifications.

Attachment Submitted filename: PONE-D-25-57279.docx

# Author comment: Author response to Decision Letter 1

**DOI:** 10.1371/journal.pone.0353938.r002

12 Feb 2026

On behalf of my co-authors, I would like to thank you for giving us an opportunity to submit a revised version of our manuscript entitled “Sensitivity of machine learning regression models to data structure and quality in crop yield prediction” to PLOS ONE. I would also like to thank you and the reviewers for the valuable time you devoted to proofreading the manuscript and for your valuable comments. Your constructive comments have been very helpful in improving the quality and clarity of our manuscript. We provide point-by-point responses to the reviewers’ comments in the .pdf file named "Response to reviewers". The comments are reproduced in italics, and our responses are provided in regular (upright) font.

Sincerely,

On behalf of Zinzinhédo, Mitchozounnou, Salako, and Glèlè Kakaï.

Attachment Submitted filename: Response to Reviewers.pdf

# Peer review: Decision Letter 1

**DOI:** 10.1371/journal.pone.0353938.r003

**Authors:** Rajib Chowdhury

3 May 2026

Dear Dr. Zinzinhédo,

Thank you for submitting your manuscript to PLOS ONE. After careful consideration, we feel that it has merit but does not fully meet PLOS ONE’s publication criteria as it currently stands. Therefore, we invite you to submit a revised version of the manuscript that addresses the points raised during the review process.

Please submit your revised manuscript by Jun 17 2026 11:59PM. If you will need more time than this to complete your revisions, please reply to this message or contact the journal office at plosone@plos.org. When you're ready to submit your revision, log on to https://www.editorialmanager.com/pone/ and select the 'Submissions Needing Revision' folder to locate your manuscript file.

- A letter that responds to each point raised by the academic editor and reviewer(s). You should upload this letter as a separate file labeled 'Response to Reviewers'.
- A marked-up copy of your manuscript that highlights changes made to the original version. You should upload this as a separate file labeled 'Revised Manuscript with Track Changes'.
- An unmarked version of your revised paper without tracked changes. You should upload this as a separate file labeled 'Manuscript'.

If you would like to make changes to your financial disclosure, please include your updated statement in your cover letter. Guidelines for resubmitting your figure files are available below the reviewer comments at the end of this letter.

If applicable, we recommend that you deposit your laboratory protocols in protocols.io to enhance the reproducibility of your results. Protocols.io assigns your protocol its own identifier (DOI) so that it can be cited independently in the future. For instructions see: https://journals.plos.org/plosone/s/submission-guidelines#loc-laboratory-protocols. Additionally, PLOS ONE offers an option for publishing peer-reviewed Lab Protocol articles, which describe protocols hosted on protocols.io. Read more information on sharing protocols at https://plos.org/protocols?utm_medium=editorial-email&utm_source=authorletters&utm_campaign=protocols.

As the corresponding author, your ORCID iD is verified in the submission system and will appear in the published article. PLOS supports the use of ORCID, and we encourage all coauthors to register for an ORCID iD and use it as well. Please encourage your coauthors to verify their ORCID iD within the submission system before final acceptance, as unverified ORCID iDs will not appear in the published article. *Only* the individual author can complete the verification step; PLOS staff *cannot* verify ORCID iDs on behalf of authors.

We look forward to receiving your revised manuscript.

Kind regards,

Rajib Chowdhury, M.Sc.; MPH

Academic Editor

PLOS One

Journal Requirements:

If the reviewer comments include a recommendation to cite specific previously published works, please review and evaluate these publications to determine whether they are relevant and should be cited. There is no requirement to cite these works unless the editor has indicated otherwise.

[Note: HTML markup is below. Please do not edit.]

Reviewers' comments:

Reviewer's Responses to Questions

**Comments to the Author**

Reviewer #2: All comments have been addressed

Reviewer #3: (No Response)

Reviewer #4: All comments have been addressed

Reviewer #5: (No Response)

**********

2. Is the manuscript technically sound, and do the data support the conclusions?

Reviewer #2: Yes

Reviewer #3: Yes

Reviewer #4: Yes

Reviewer #5: Yes

**********

3. Has the statistical analysis been performed appropriately and rigorously?

Reviewer #2: Yes

Reviewer #3: Yes

Reviewer #4: Yes

Reviewer #5: Yes

**********

4. Have the authors made all data underlying the findings in their manuscript fully available?

The PLOS Data policy

Reviewer #2: Yes

Reviewer #3: Yes

Reviewer #4: Yes

Reviewer #5: No

**********

5. Is the manuscript presented in an intelligible fashion and written in standard English?

Reviewer #2: Yes

Reviewer #3: Yes

Reviewer #4: Yes

Reviewer #5: Yes

**********

Reviewer #2: 1. The study is well-structured with a large simulation component and a smaller practical validation component. Could you elaborate on how the practical validation results (where kNN and XGBoost were optimal in some cases) inform or challenge the main conclusion from the simulation study (which points to the general robustness of Random Forest)?

2. The introduction effectively surveys various preprocessing techniques. Could you briefly elaborate on the rationale for selecting mean, median, MICE-Lasso, and MICE-RF for this study over other mentioned methods like k-nearest neighbor (kNN) imputation or linear interpolation?

3. You briefly draw a parallel to challenges in health science, specifically diabetes prediction. Could you expand on this comparison to better contextualize the novelty of your work? How do the data challenges in that domain reinforce the need for this specific study in agriculture?

4. For the machine learning models, could you provide more detail on the hyperparameter selection process? The manuscript mentions an "automated grid search procedure," which is appropriate, but detailing the range of parameters explored for each algorithm would significantly improve the reproducibility of your work.

5. The manuscript mentions evaluating models through cross-validation in the introduction, but the methods section describes a repeated 70/30 training/testing split iterated five times. Could you please clarify the terminology and procedure to ensure there is no ambiguity? Was this a repeated random sub-sampling validation?

6. The logarithm of the Mean Absolute Error (logMAE) is used as a performance metric. Could you explain in the methods the reason for this transformation and how it should be interpreted by the reader, particularly in contrast to the more standard MAE?

7. The number of retained Principal Components (PCs) was set at 2, 3, and 5. What was the rationale for choosing these specific numbers? Were they based on a common heuristic, such as a certain threshold of explained variance, or were they chosen to represent different levels of dimensionality reduction?

8. One of your most intriguing findings is the "multicollinearity paradox," where high multicollinearity inflated R² while also worsening MAE. Could you expand on this in the discussion section? What is the underlying statistical mechanism that causes this apparent paradox, and how does it relate to model overfitting?

9. In Table 4, the results suggest that while Random Forest (RF) often has a higher R² on small samples, other models like SVM and kNN can achieve a lower MAE on larger samples. Your conclusion is that "RF is the most robust algorithm." Could you refine this statement to reflect the nuance that the "best" model might depend on whether the primary goal is maximizing explained variance (R²) or minimizing prediction error (MAE)?

10. The practical validation in Table 9 shows that the optimal workflow for Peanuts (high collinearity, no missingness) was using 2 PCs with an XGBoost model. How does this finding support or modify your general conclusion that MICE-regression paired with 2 PCs is the most reliable workflow?

11. The study provides an excellent analysis of model accuracy and error. Could you add a brief discussion on the computational costs associated with the different imputation methods and algorithms? A qualitative analysis of the trade-off between performance gains and computational expense would be very valuable for practitioners.

12. The conclusion effectively summarizes the key takeaways. Could you extend it to provide a more prescriptive set of guidelines for researchers? For example, based on your findings, what workflow would you recommend to a researcher who has a small dataset with moderate multicollinearity and a high percentage of missing values?

Reviewer #3: Summary:

The revised manuscript presents a comprehensive simulation-based analysis of the sensitivity of machine learning regression models to data structure, imperfections, and preprocessing strategies in the context of crop yield prediction. The authors have made substantial improvements in response to previous reviewer comments, including the integration of real-world datasets, inclusion of additional machine learning models (XGBoost, LightGBM, Neural Networks), incorporation of advanced imputation methods (MICE-based techniques), and the addition of post-hoc statistical testing. Overall, the manuscript is significantly strengthened and provides valuable insights into ML robustness under imperfect data conditions.

Major Comments:

The authors have adequately addressed most of the major concerns raised in the previous review round. In particular:

- The inclusion of real-world datasets improves the practical relevance and generalizability of the study.

- The expansion of the model set to include modern state-of-the-art algorithms is appropriate and well-integrated.

- The addition of Tukey HSD post-hoc analysis strengthens the statistical rigor.

- The methodology is now more clearly described, including hyperparameter tuning and validation strategy.

Minor Comments:

1. Abstract inconsistency:

The Abstract contains an inconsistency regarding the number of machine learning models. While a specific number is mentioned, the listed models do not fully align with that count. Please revise the Abstract to ensure consistency between the stated number and the models included in the study.

2. wording issue in Results:

In the Results section (around line 252), the authors state that Random Forest (RF) “achieved the lowest” performance, whereas the context and reported values in (table 5) indicate that RF actually achieved the highest performance. Please correct this wording to accurately reflect the results.

3. Clarity on validation strategy:

While the revised manuscript explains repeated random sub-sampling and 5 fold cross validation, the wording may still cause slight confusion. Consider clarifying the distinction between these procedures to improve readability.

Conclusion:

The manuscript is technically sound, methodologically improved, and relevant to the field. Only minor revisions related to clarity, consistency, and language are required before publication.

Recommendation: Minor Revision

Reviewer #4: 1)The integration of an empirical dataset to evaluate performance introduces significant novelty to this work. To ensure the broader impact of the framework are immediately clear, this contribution should be mentioned in the Abstract. Doing so will clarify for the reader that the findings extend beyond simulated environments. Additionally, within the 'Validation through Practical Crop Datasets' subsection, the authors should address the potential for performance variability, stating how the pipeline's robustness might be affected when applied to different or more diverse datasets.

2)The methodology/resource regarding the empirical data characteristics requires further clarification. Currently, it is unclear how the specific attributes(Low missingness, No missingness) of the data were identified or achieved. Please provide a more transparent description of this process to support the technical rigor of the study and to ensure that the results are fully reproducible by other researchers.

3) While the content is logically sound, the use of 'Practice' as a heading/subheading is too vague and fails to convey the conceptual focus of the subsection (I recommend replacing it with a more descriptive title such as 'Practical Implementation' ).

4) Furthermore, the paragraphs in Results section appear fragmented due to a lack of consistent heading and subheading conventions. Please adopt a standardized hierarchy, similar to the formatting used in the 'Methodology' section to ensure structural coherence throughout the manuscript.

Reviewer #5: The authors have addressed many of the major concerns raised in the first review round, especially by expanding the model set, adding empirical crop datasets, strengthening the statistical analysis, and improving data/code availability. However, several reviewer/editor concerns are only partially addressed, and a few new inconsistencies introduced during revision should be corrected before the manuscript is accepted.

1. The Abstract states that the study evaluated “RF, SVM, MLR, XGBoost, LightBoost, and kNN,” which lists only six algorithms and omits Neural Networks, although the manuscript later states that seven algorithms were used. It also uses “LightBoost,” whereas the Methods use Light Gradient Boosting Machine / LGBM. This should be corrected throughout.

2. The manuscript states that 1,728 combinations were run for each ML algorithm and that the seven algorithms produced 12,096 scenarios in total. However, the Abstract says “1,728 scenarios across the seven ML algorithms,” which underreports the total experimental runs. The authors should clearly distinguish between: “1,728 data/pre-processing scenarios per algorithm” and “12,096 model-scenario evaluations in total.”

3. Hyperparameter tuning needs more detail. The authors should add a supplementary table listing the tuned hyperparameters and ranges for each model, for example: RF mtry, number of trees; SVM C, sigma; kNN k; XGBoost eta, max_depth, nrounds; LGBM parameters; and NNet hidden units/decay. This is important for reproducibility.

4. The manuscript would benefit from a broader regression-evaluation framework. Although R² and MAE are useful, the authors should consider adding MAPE, as it provides an intuitive percentage-based measure of prediction error and facilitates comparison across crops and yield scales. RMSE or normalized RMSE could also be reported to strengthen the performance comparison.

5. Since “sensitivity” is central to the manuscript title, the sensitivity analysis should be strengthened. In addition to missingness, multicollinearity, sample size, and predictor number, the authors should consider testing model robustness under controlled perturbations such as Gaussian noise injection into the input predictors. The manuscript should also acknowledge uncertainty-aware approaches, such as quantile regression evaluated with pinball loss, either as an additional analysis or as a clear direction for future work.

6. A dedicated section titled “Limitations and Future Work” should be added before the Conclusion. This section should clearly discuss the main limitations of the study, including the reliance on simulated data, the limited number of real-world crop datasets, the selected missingness mechanism, the restricted set of algorithms, and the limited evaluation metrics.

**********

what does this mean?). If published, this will include your full peer review and any attached files.

If you choose “no”, your identity will remain anonymous but your review may still be made public.

**Do you want your identity to be public for this peer review?** For information about this choice, including consent withdrawal, please see our Privacy Policy

Reviewer #2: No

Reviewer #3: No

Reviewer #4: No

Reviewer #5: **Yes:** Sarowar Morshed Shawon

**********

[NOTE: If reviewer comments were submitted as an attachment file, they will be attached to this email and accessible via the submission site. Please log into your account, locate the manuscript record, and check for the action link "View Attachments". If this link does not appear, there are no attachment files.]

To ensure your figures meet our technical requirements, please review our figure guidelines: https://journals.plos.org/plosone/s/figures

You may also use PLOS’s free figure tool, NAAS, to help you prepare publication quality figures: https://journals.plos.org/plosone/s/figures#loc-tools-for-figure-preparation.

NAAS will assess whether your figures meet our technical requirements by comparing each figure against our figure specifications.

# Author comment: Author response to Decision Letter 2

**DOI:** 10.1371/journal.pone.0353938.r004

10 Jun 2026

On behalf of my co-authors,

I would like to thank the Editor and the Reviewers for the valuable time devoted to evaluate the manuscript and for their valuable comments. Their constructive comments have been very helpful in improving the quality and clarity of our manuscript. We provide point-by-point responses to the reviewers’ comments in the file labeled 'Response to Reviewers'. The comments are reproduced in italics, and our responses are provided in regular (upright) font.

Sincerely,

On behalf of Zinzinhédo, Mitchozounnou, Salako, and Glèlè Kakaï

Attachment Submitted filename: Response_to_Reviewers_auresp_2.pdf

# Peer review: Decision Letter 2

**DOI:** 10.1371/journal.pone.0353938.r005

**Authors:** Rajib Chowdhury

1 Jul 2026

Sensitivity of machine learning regression models to data structure and quality in crop yield prediction.

PONE-D-25-57279R2

Dear Dr. Zinzinhédo,

We’re pleased to inform you that your manuscript has been judged scientifically suitable for publication and will be formally accepted for publication once it meets all outstanding technical requirements.

Within one week, you’ll receive an e-mail detailing the required amendments. When these have been addressed, you’ll receive a formal acceptance letter and your manuscript will be scheduled for publication.

An invoice will be generated when your article is formally accepted. Please note, if your institution has a publishing partnership with PLOS and your article meets the relevant criteria, all or part of your publication costs will be covered. Please make sure your user information is up-to-date by logging into Editorial Manager at Editorial Manager® and clicking the ‘Update My Information' link at the top of the page. For questions related to billing, please contact billing support.

If your institution or institutions have a press office, please notify them about your upcoming paper to help maximize its impact. If they’ll be preparing press materials, please inform our press team as soon as possible -- no later than 48 hours after receiving the formal acceptance. Your manuscript will remain under strict press embargo until 2 pm Eastern Time on the date of publication. For more information, please contact onepress@plos.org.

Kind regards,

Rajib Chowdhury, M.Sc.; MPH

Academic Editor

PLOS One

Additional Editor Comments (optional):

Reviewers' comments:

Reviewer's Responses to Questions

**Comments to the Author**

Reviewer #2: All comments have been addressed

Reviewer #4: All comments have been addressed

**********

2. Is the manuscript technically sound, and do the data support the conclusions?

Reviewer #2: Yes

Reviewer #4: Yes

**********

3. Has the statistical analysis been performed appropriately and rigorously?

Reviewer #2: Yes

Reviewer #4: Yes

**********

4. Have the authors made all data underlying the findings in their manuscript fully available?

The PLOS Data policy

Reviewer #2: Yes

Reviewer #4: Yes

**********

5. Is the manuscript presented in an intelligible fashion and written in standard English?

Reviewer #2: Yes

Reviewer #4: Yes

**********

Reviewer #2: The manuscript has been improved significantly. The authors have addressed all my concerns and responded to my review satisfactorily. I would recommend to accept this manuscript in the present form.

Reviewer #4: The paper's quality has significantly improved from a reader's perspective, as all previous comments have been thoroughly addressed.

However, it is important to recheck for typos before the final submission; for example, a clear error in "Sensitivit" remains on line 282.

**********

what does this mean?). If published, this will include your full peer review and any attached files.

If you choose “no”, your identity will remain anonymous but your review may still be made public.

**Do you want your identity to be public for this peer review?** For information about this choice, including consent withdrawal, please see our Privacy Policy

Reviewer #2: No

Reviewer #4: No

**********

# Editor report: Acceptance letter

**DOI:** 10.1371/journal.pone.0353938.r006

**Authors:** Rajib Chowdhury

PONE-D-25-57279R2

PLOS One

Dear Dr. Zinzinhédo,

I'm pleased to inform you that your manuscript has been deemed suitable for publication in PLOS One. Congratulations! Your manuscript is now being handed over to our production team.

At this stage, our production department will prepare your paper for publication. This includes ensuring the following:

* All references, tables, and figures are properly cited

* All relevant supporting information is included in the manuscript submission,

* There are no issues that prevent the paper from being properly typeset

You will receive further instructions from the production team, including instructions on how to review your proof when it is ready. Please keep in mind that we are working through a large volume of accepted articles, so please give us a few days to review your paper and let you know the next and final steps.

Lastly, if your institution or institutions have a press office, please let them know about your upcoming paper now to help maximize its impact. If they'll be preparing press materials, please inform our press team within the next 48 hours. Your manuscript will remain under strict press embargo until 2 pm Eastern Time on the date of publication. For more information, please contact onepress@plos.org.

You will receive an invoice from PLOS for your publication fee after your manuscript has reached the completed accept phase. If you receive an email requesting payment before acceptance or for any other service, this may be a phishing scheme. Learn how to identify phishing emails and protect your accounts at https://explore.plos.org/phishing.

If we can help with anything else, please email us at customercare@plos.org.

Thank you for submitting your work to PLOS One and supporting open access.

Kind regards,

PLOS ONE Editorial Office Staff

on behalf of

Dr. Rajib Chowdhury

Academic Editor

PLOS One
